# Endophytic bacteria in *Camellia reticulata* pedicels: isolation, screening and analysis of antagonistic activity against nectar yeasts

**DOI:** 10.3389/fmicb.2024.1459354

**Published:** 2024-10-21

**Authors:** Qingxin Meng, Rong Huang, Lijie Xun, Xiaoman Wu, Shangkao Deng, Dan Yue, Wenzheng Zhao, Xia Dong, Xueyang Gong, Kun Dong

**Affiliations:** ^1^Yunnan Provincial Engineering and Research Center for Sustainable Utilization of Honey Bee Resources, Eastern Bee Research Institute, College of Animal Science and Technology, Yunnan Agricultural University, Kunming, China; ^2^Institute of Sericulture and Apiculture, Yunnan Academy of Agricultural Sciences, Mengzi, China; ^3^College of Food Science and Technology, Yunnan Agricultural University, Kunming, China

**Keywords:** tree age, pedicel endophytic bacteria, anti-yeast potential, bioactive metabolites, antibiotics, nectar homeostasis

## Abstract

*Camellia reticulata*, an ancient plant species endemic to Yunnan Province, China, remains underexplored in terms of its endophytic bacterial communities. The plant tissue pedicel serves as the connection between the flower and the stem, not only delivers nutrients but also transmits metabolic substances from endophytic bacteria to the nectar during long-term microbial colonization and probably improves the antagonistic activity of nectar against yeast. Hence, 138 isolates of endophytic bacteria have been isolated in this study from the pedicels of 12- and 60-year-old *C. reticulata*. Comparative analysis revealed significantly higher density of endophytic bacteria in older trees. Among these isolates, 29 exhibited inhibitory effects against nectar yeasts. Most of the isolates displayed positive results for Gram staining, catalase reaction, gelatin liquefaction, and motility. Additionally, the isolates demonstrated the ability to utilize diverse substrates, such as glucose, nitrate, and starch. Based on 16S rRNA molecular biology analysis, these isolates were identified to be 11 different species of 6 genera, with the majority belonging to *Bacillus* genus. Notably, C1 isolate, identified as *Bacillus spizizenii*, exhibited strongest antagonistic effect against three yeasts, i.e., *Metschnikowia reukaufii, Cryptococcus laurentii*, and *Rhodotorula glutinis*, with minimum inhibitory concentration values below 250 μg/mL. Major metabolites of *B. spizizenii* were aminoglycosides, beta-lactams, and quinolones, which possess antimicrobial activities. Furthermore, KEGG enrichment pathways primarily included the synthesis of plant secondary metabolites, phenylpropanoids, amino acids, alkaloids, flavonoids, neomycin, kanamycin, and gentamicin. Therefore, antagonistic activity of *B. spizizenii* against yeasts could be attributed to these antibiotics. The findings highlight the diverse endophytic bacteria associated with *C. reticulata*, indicating their potential as a valuable resource of bioactive metabolites. Additionally, this study provides new insights into the role of endophytic bacteria of pedicels in enhancing nectar resistance against yeasts.

## 1 Introduction

Nectar is a liquid secreted by the nectaries of flowering plants. It is rich in sugars, amino acids, organic acids, volatile substances, and various other compounds, attracting pollinators (bees, butterflies, birds, and other animals) and aiding in pollination (Canto and Herrera, 2012). However, the pollinators can pose a risk of nectar contamination by yeasts and bacteria (Russell et al., 2019; Zemenick et al., 2021). In particular, the nectar rich in various nutrients is highly conducive to microbial growth and proliferation. Yeasts belonging to phyla Ascomycota and Basidiomycota are found commonly in nectar, including genera *Metschnikowia, Cryptococcus, Rhodotorula*, and *Saccharomyces* (Canto and Herrera, 2012; Zhou et al., 2016). These yeasts have a unique ecological niche, as they live and proliferate in the nectar of flowers and can significantly alter the composition and concentration of sugars and amino acids, reducing the total sugar concentration and transforming the sucrose-rich nectar into fructose-rich nectar, thus affecting the interactions between plant and pollinators (Canto and Herrera, 2012; Zhou et al., 2016). It is obvious that the nectar does not decay due to microbial contamination and its quality remains superior, which indicates the presence of an effective antimicrobial mechanism (Herrera et al., 2008, 2009; Canto and Herrera, 2012). However, research on the antimicrobial substances in floral nectar is still in the exploratory stage. Furthermore, numerous factors may contribute to the efficacy of these substances, which need to be explored (Sasu et al., 2010).

Endophytic bacteria form a symbiotic relationship with the plant. They live inside plant tissues without causing any harm to host, providing resistance to plant against disease and environmental stress, promoting plant growth, and inhibiting the pathogens (Santoyo et al., 2016). In recent years, endophytic bacteria have been utilized as biocontrol agent to suppress plant pathogens. They can produce various antimicrobial metabolites, including alkaloids, peptides, steroids, and terpenoids, thereby providing resistance to host plants against microbial stress (Zhang et al., 2006; Pieterse et al., 2014). For instance, *Bacillus velezensis* KOF112 exhibited broad-spectrum antifungal activity and contributed to disease prevention and control in grape cultivation in a previous study (Hamaoka et al., 2021). Metabolites secreted by *Sphingomonas melonis*, isolated from rice seeds, exhibited significant antagonistic effects against the plant pathogen *Burkholderia plantarii* (Matsumoto et al., 2021). In addition, metabolites of *Bacillus thuringiensis*, isolated from *Cordia dichotoma*, also displayed diverse antimicrobial activities (Sharma and Mallubhotla, 2022). These reports highlight the significant role of endophytic bacteria in helping host plants respond to microbial infections. The colonization of endophytes can alter the composition of volatile metabolites and microbiota in nectar, which in turn attracts more pollinators and enhances the reproductive fitness of plants (González-Mas et al., 2023). These findings provide a new research avenue for endophytic bacteria to assist nectar resistance to yeasts.

*Camellia reticulata* (Theaceae family) is a rare and protected plant species in China. It is a unique woody oil tree species in Yunnan Province, which has a long history of cultivation by local people and holds great significance in economy (Feng, 1980). Observably, *C. reticulata* with the large quantity of nectar produced by its flowers over a long flowering period, experiences frequent visitation by birds, making the nectar highly susceptible to yeast contamination. However, its nectar is not fermented quickly in such a process. In previous studies, endophytic bacteria have been found in the roots, stems, and leaves of *C. reticulata*, exhibiting antagonistic effects against root rot, leaf blight, anthracnose, and soft rot (Li et al., 2009; Jiang et al., 2016). This shows that the plant tissue of *C. reticulata* is rich in endophytic bacteria, and improves the plant's fitness. Pedicels are crucial components of flowers, supporting not only their spatial distribution but also serving as the channels that connect the stems and flowers and enable the transport of nutrients from plant to flowers (Zhou, 2015). The sap, produced in the phloem sieve tubes of plants, is the primary component required for nectar formation, which is transported to the nectaries through the sieve tubes (Tejesvi et al., 2013). The endophytic bacteria are closely associated with this process, as they colonize the plant pedicels for extended periods and serve as valuable biological resources. However, there has been limited research on endophytic bacteria living in the pedicels of *C. reticulata* and their antagonistic activity against nectar yeasts. This research focuses on: (i) isolating endophytic bacteria from *C. reticulata* pedicels, (ii) screening and identifying antagonistic isolates against nectar yeasts, (iii) evaluating *Bacillus spizizenii*'s anti-yeast potential, and analyzing its metabolite composition. The aim is to understand how heterospecific-pollinated plants maintain the stability and quality of nectar under yeast stress.

## 2 Materials and methods

### 2.1 Collection of plant samples

**Figure 1:**
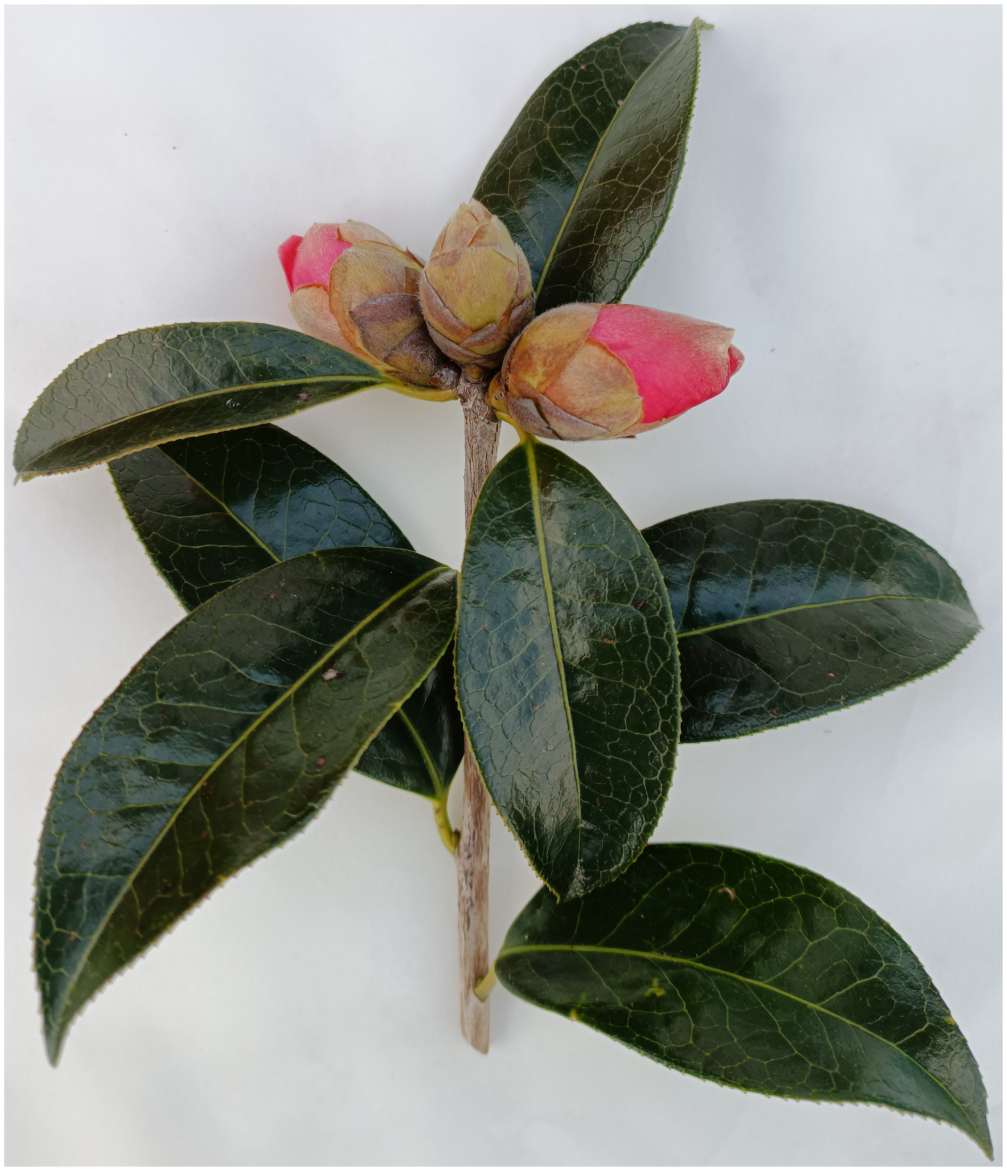
A flower bud sample of *C. reticulata* at fully closed calyx stage.

In February 2023, samples of *C. reticulata* were collected from Shaba Forest Farm in Tengchong, Yunnan Province (98°5′86″ E, 24°9′57″ N). This area has a tropical monsoon climate, with an average annual temperature of 14.6°C, average sunshine duration of ~2,167 h, average precipitation of 1,531 mm, and average altitude of 2,149 m. Based on the tree information management archival records of the forest farm, 12- and 60-year-old healthy *C. reticulata* plants were selected for sample collection. Specifically, fresh flower buds at the fully closed calyx stage were randomly collected from *C. reticulata* using sterilized scissors, placed in sterile sampling bags, and stored at 4°C (Figure 1).

### 2.2 Isolation of endophytic bacteria

#### 2.2.1 Surface sterilization of samples

After washing the samples under flowing tap water, flower buds and stems were removed under sterile conditions, while preserving the pedicels. Following the method described by Cardoso et al. (2020), rigorous surface sterilization of pedicels was performed. Firstly, the pedicels were rinsed with sterile distilled water, and then immersed in 75% ethanol for 60 s. After four repeated rinsing steps, pedicels were dried using sterile filter paper, and then immersed in a 5% NaClO solution for 2 min. Subsequently, pedicels were rinsed again four times with sterile distilled water and dried with sterilized filter paper. To test the effectiveness of surface sterilization, 100 μL of the final rinse water was spread onto Luria Bertani (LB) and Tryptic Soy Agar (TSA) culture media. No microbial growth on media after 72 h incubation at 30°C indicated successful surface sterilization of the pedicels.

#### 2.2.2 Isolation, purification, and preservation of endophytic bacteria

Firstly, 1 g surface-sterilized pedicels were transferred into a sterile mortar and grounded along with a small amount of quartz sand and 1 mL of PBS solution. After thorough grinding, 9 mL of PBS solution was added into the pedicel extracts and mixed well. Subsequently, 10^1^-10^4^-fold dilution gradients were prepared, and 100 μL of these diluted suspensions were plated onto both LB and TSA culture media. The culture plates were then incubated at 30°C for 24–72 h, with three replicates for each diluted suspension. LB agar medium, exhibiting superior results compared to TSA medium, was selected for further isolation and purification of endophytic bacteria. Based on colony morphology, isolates were purified through repeated streaking and sub-culturing. The purified isolates were cultured in LB broth media at 30°C and 130 rpm until they reached the exponential growth phase. Finally, pure isolates were preserved in 50% glycerol stocks and stored at −80°C for subsequent experiments.

### 2.3 Screening of endophytic bacteria with antagonistic activity against nectar yeasts

Three yeasts were used as indicators to screen the endophytic bacteria with antagonistic activity against nectar yeasts. These yeasts, *Metschnikowia reukaufii* (CGMCC2.2728), *Cryptococcus laurentii* (CGMCC2.2425), and *Rhodotorula glutinis* (CGMCC2.2945), were all procured from the China General Microbiological Culture Collection Center (CGMCC).

Firstly, the yeasts were recovered and cultured using Malt Extract Broth (MEB) medium. Their suspensions were prepared by diluting the cultures to a concentration of 0.5 McFarland standard (~10^8^ CFU/mL) using PBS solution. A sterile cotton swab was used to evenly spread the prepared suspensions onto Potato Dextrose Agar (PDA) plates as the test media plates. Subsequently, single colonies of endophytic bacterial isolates isolated from the *C. reticulata* pedicels were picked with an inoculating needle and placed on the test media plates. After overnight incubation at 28°C, inhibition zones on the plates were observed. This step is a preliminary screening, which is simple to operate and can quickly find endophytic bacteria with antagonistic effect on nectar yeast. To evaluate their anti-yeast effects, a re-screening using equivalent quantities of fermentation broth is required. These bacteria were then cultured in LB broth media for 3 days at 34°C with shaking at 120 r/min. The supernatants were obtained by centrifugation of cultures at 12,000 rpm for 15 min, and used to determine the antagonistic activity against nectar yeasts. Hundred microliter of supernatant was added to the oxford cups, while LB medium without the supernatant of endophytic bacteria served as a negative control. LB medium with amphotericin B was used as a positive control. All media were sealed with parafilm and incubated at 28°C for 24 h. After incubation, diameter of inhibition zone produced by each endophytic bacterial isolate against yeasts were measured.

### 2.4 Identification of endophytic bacteria with antagonistic activity against nectar yeasts

The endophytic bacterial isolate showing antagonistic activity against yeasts were preliminarily identified by observing their morphological characteristics, Gram-reaction, motility, and activities of amylase, catalase, and gelatinase enzymes (Goodfellow et al., 2009). In addition, using the total DNA extraction kit (E.Z.N.A.^®^ Soil DNA Kit), DNA of each endophytic bacterial isolate with anti-yeast activity was extracted. A NanoDrop spectrophotometer was used to determine the concentration of genomic DNA by measuring the absorbance at 260 nm. The primers 8F (5′-AGAGTTTGATCCTGGCTCAG-3′) and 1492R (5′-GGTTACCTTGTTACGACTT-3′) were used to amplify the 16S rRNA gene of isolated DNA samples through polymerase chain reaction (PCR). Due to their high conservativeness, broad applicability, moderate GC content and Tm values, as well as strong support in the literature, have become the preferred primer pair for the amplification and identification of bacterial 16S rRNA genes, and are widely used for bacterial identification. The reaction system (25 μL) consisted of: 1 μL of each primer (10 μmol/L), 2 μL of template (20 ng/μL), and 21 μL of PCR Mix. PCR reaction conditions were set as: 96°C for 5 min; followed by 35 cycles of 96°C for 20 s, 56°C for 30 s, and 72°C for 30 s; and a final extension at 72°C for 10 min. PCR products were stored at 4°C. The amplification results were verified by 1% agarose gel electrophoresis (150 V/100 mA for 20 min), and the target DNA fragments were recovered using the QIAquick Agarose Gel Recovery Kit (Item code: 28704). Purified DNA fragments were sent to Shanghai Majorbio Bio-Pharm Technology Co., Ltd. for sequencing. The obtained sequences were aligned using DNAMAN 6.0.3.99 (Lynnon Biosoft, San Ramon, CA, USA) and analyzed using BlastN program (http://www.ncbi.nlm.nih.gov/BLAST). The sequences were also cross-checked with the EzTaxon and RDP databases to select the highest scoring species for further analysis, based on the similarity to identified bacteria. All obtained bacterial 16S rRNA sequences have been deposited in GenBank (Supplementary Table 1).

### 2.5 Extraction of crude extract of *B. spizizenii* with ethyl acetate

The endophytic bacterium C1 *B. spizizenii* was activated and revitalized prior to inoculation into LB broth medium. It was then incubated at 34°C in a shaking incubator at 120 r/min for 12 h to obtain the seed bacterial culture. Subsequently, a 2% inoculum of the seed culture was added to fresh LB broth and cultured at 34°C with shaking at 120 r/min for 3 days to produce an abundant fermentation broth. The fermentation broth was then subjected to ultrasonic disruption for 1 h, followed by UV light irradiation for 45 min. Thereafter, it was centrifuged at 12,000 r/min for 15 min to collect the supernatant. Ethyl acetate was used to extract the supernatant in a 1:1 volume ratio. The resulting solution was rotary evaporated at 45°C to remove the ethyl acetate, yielding a crude extract that was stored at 4°C for future use.

### 2.6 Analysis of the anti-yeast properties of metabolites from *B. spizizenii*

The method described by Wang et al. (2023) was employed to analyze the antimicrobial properties of ethyl acetate extract obtained from the endophytic bacterium *B. spizizenii*. The minimum inhibitory concentration (MIC) values were determined using the broth dilution method in accordance with the guidelines of Clinical and Laboratory Standards Institute (CLSI), using amphotericin B as the positive control. Specifically, in each well of the first row of a 96-well plate, 180 μL of yeast suspension (1.25 × 10^6^ CFU/mL) was added, while the remaining wells received only 100 μL of yeast suspension. A crude extract solution of *B. spizizenii* in DMSO (10 mg/mL) was prepared, and 20 μL of this solution was added into the wells of first row containing 180 μL of yeast suspension. Subsequently, a 2-fold serial dilution was performed within the 96-well plate, and the turbidity in each well was observed to determine the lowest concentration of *B. spizizenii* required to inhibit yeast growth. This lowest concentration was considered the MIC value.

### 2.7 Determination of the components of metabolites obtained from *B. spizizenii*

Non-targeted metabolomics liquid chromatography and mass spectroscopy (LC-MS/MS) was used to analyze the metabolites of *B. spizizenii*. A sample of crude extract weighing 20 mg (±1 mg) was added to 400 μL of a 70% methanol-water internal standard extraction solution. The mixture was vortexed for 3 min and subjected to ultrasonic extraction in an ice-water bath for 10 min. After removing the sample, it was vortexed for an additional 1 min and allowed to stand at −20°C for 30 min. Following this, the mixture was centrifuged at 12,000 r/min for 10 min at 4°C, and the resulting supernatant was filtered through a 0.22 μm membrane. The filtered solution was then transferred to a detection vial for LC-MS/MS analysis. This process was conducted at Shanghai Majorbio Bio-Pharm Technology Co., Ltd. The extracted metabolites were then subjected to analysis of pathways using the Kyoto Encyclopedia of Genes and Genomes (KEGG) database. The molecular weights of the metabolites were determined based on the m/z ratios of the parent ions in the MS1 spectra. Molecular formula predictions were made using mass deviation (ppm) and adduct ion information, followed by matching with the database for primary identification of the metabolites. Additionally, for quantitative analysis, the MS2 spectra of the detected metabolites were compared with fragment ion information from the database to achieve secondary identification.

### 2.8 Statistical analysis

SPSS 22 statistical software (SPSS Inc., Chicago, IL, USA) was used to calculate the mean and standard deviation of the bacterial densities and the size of inhibition zones produced due to anti-yeast activity of endophytic bacteria. *P* < 0.05 indicates a statistically significant difference.

## 3 Results

### 3.1 Isolation of endophytic bacteria from pedicels of *C. reticulata*

Disinfection is crucial to remove the unwanted microorganisms from the surface of plant samples. After 72 h tissue culturing, no microbial growth was observed on LB and TSA media. This confirmed the successful surface sterilization of collected pedicels of *C. reticulata*. Based on colony morphology, a total of 138 endophytic bacterial isolates were isolated and purified from the pedicels of 12- and 60-year-old *C. reticulata*, with bacterial densities of 3.9 × 10^3^ ± 0.72 × 10^3^ CFU/g and 16.5 × 10^3^ ± 3.58 × 10^3^ CFU/g, respectively. Among the 138 isolates, 52 were isolated from 12-year-old trees and 86 were isolated from 60-year-old trees.

### 3.2 Screening of endophytic bacteria with antagonistic activity against nectar yeasts

These isolates were subjected to preliminarily screening for antagonistic activity against nectar yeasts. Around 21.01% isolates (29 isolates) showed inhibitory effects on at least one yeast, with 20 inhibiting *M. reukaufii*, 24 inhibiting *C. laurentii*, and 27 inhibiting *R. glutinis*. Meanwhile, 11.59% isolates (16 isolates) exhibited antagonistic activity against all three yeasts, including C1, C37, C38, C46, F1, F2, F6, F9, F15, F22, F37, F44, F50, F51, F53, and F56 (Table 1).

**Table 1:** Screening of endophytic bacteria isolated from the pedicels of *C. reticulata* with antagonistic activity against nectar yeasts.

**Isolates**	***Metschnikowia reukaufii***	***Cryptococcus laurentii***	***Rhodotorula glutinis***
C1	+	+	+
C3	+	+	–
C9	–	–	+
C10	–	–	+
C18	–	–	+
C37	+	+	+
C38	+	+	+
C46	+	+	+
C50	–	–	+
F1	+	+	+
F2	+	+	+
F4	–	+	+
F6	+	+	+
F9	+	+	+
F12	–	+	+
F13	–	+	+
F14	–	+	+
F15	+	+	+
F22	+	+	+
F36	–	+	+
F37	+	+	+
F40	+	+	–
F44	+	+	+
F45	+	+	–
F46	–	–	+
F50	+	+	+
F53	+	+	+
F56	+	+	+
F60	+	–	+

“+” represents the presence of antagonistic activity against nectar yeast, while “–” represents the absence of such activity.

Furthermore, the antagonistic activity of isolates against yeasts was further assessed by the diameter of their inhibition zones produced by their fermentation substances (Table 2). The screening results showed the antagonistic activity of 29 isolates against at least one yeast. Except for F45, all isolates demonstrated antagonistic effects against *R. glutinis*. Among these, C1 showed the best inhibitory effect against all three yeasts, similar to amphotericin B, highlighting its significant anti-yeast potential. The fermentation substances of C1 produced inhibition zones with diameters of 19.23 ± 0.95 mm, 19.93 ± 0.68 mm, and 21.53 ± 1.13 mm against *M. reukaufii, C. laurentii*, and *R. glutinis*, respectively.

**Table 2:** Diameter of inhibition zones of endophytic bacteria with antagonistic activity against nectar yeasts (mean ± S.E., unit: mm).

**Isolates**	***Metschnikowia reukaufii***	***Cryptococcus laurentii***	***Rhodotorula glutinis***
C1	19.23 ± 0.95	19.93 ± 0.68	21.53 ± 1.13
C3	11.83 ± 0.29	12.30 ± 0.51	11.13 ± 0.34
C9	15.03 ± 0.45	8.80 ± 0.57	15.73 ± 0.56
C10	10.43 ± 0.63	10.47 ± 0.82	15.07 ± 0.42
C18	–	–	15.87 ± 0.45
C37	10.43 ± 0.49	10.83 ± 0.24	9.60 ± 0.14
C38	9.93 ± 0.42	11.53 ± 2.67	9.93 ± 0.19
C46	9.47 ± 0.47	8.80 ± 0.42	10.80 ± 0.57
C50	–	–	15.57 ± 0.54
F1	10.67 ± 0.46	8.67 ± 0.39	9.30 ± 0.36
F2	10.80 ± 0.43	8.93 ± 0.68	9.07 ± 0.33
F4	–	8.30 ± 0.37	8.23 ± 0.21
F6	11.83 ± 0.50	12.83 ± 0.54	14.30 ± 0.51
F9	9.37 ± 0.33	9.60 ± 0.45	9.53 ± 0.12
F12	–	8.97 ± 0.46	8.03 ± 0.17
F13	6.87 ± 0.29	15.53 ± 0.69	11.13 ± 0.46
F14	7.20 ± 0.49	8.27 ± 0.17	8.50 ± 0.22
F15	9.33 ± 0.49	11.53 ± 0.61	9.03 ± 0.17
F22	9.63 ± 0.54	13.60 ± 0.83	8.10 ± 0.45
F36	11.07 ± 0.42	13.57 ± 0.68	8.13 ± 0.26
F37	11.57 ± 0.68	12.73 ± 0.56	12.07 ± 0.42
F40	8.23 ± 0.42	8.30 ± 0.36	9.63 ± 0.42
F44	9.00 ± 0.16	8.30 ± 0.16	8.50 ± 0.08
F45	8.40 ± 0.37	9.67 ± 0.39	–
F46	8.53 ± 0.53	–	8.3 ± 0.12
F50	9.77 ± 0.52	9.07 ± 0.40	7.97 ± 0.17
F53	15.43 ± 0.74	15.00 ± 0.59	11.17 ± 0.29
F56	8.13 ± 0.05	7.63 ± 0.12	11.37 ± 0.54
F60	11.77 ± 0.54	–	9.20 ± 0.24
LB mediunm	–	–	–
Amphotericin B	24.30 ± 3.48	20.27 ± 0.88	21.73 ± 0.34

“–” indicates no inhibitory activity against nectar yeast.

Additionally, spot inoculation with isolates C3, C9, C10, F13, F14, and F46 initially resulted in no antagonistic effects against some yeasts. However, the fermentation products of these isolates exhibited noticeable inhibitory effects on the corresponding yeasts. Therefore, it is necessary to use various methods to evaluate the antagonistic activity of endophytic bacteria against nectar yeasts.

### 3.3 Characterization of screened endophytic bacterial isolates

**Figure 2:**
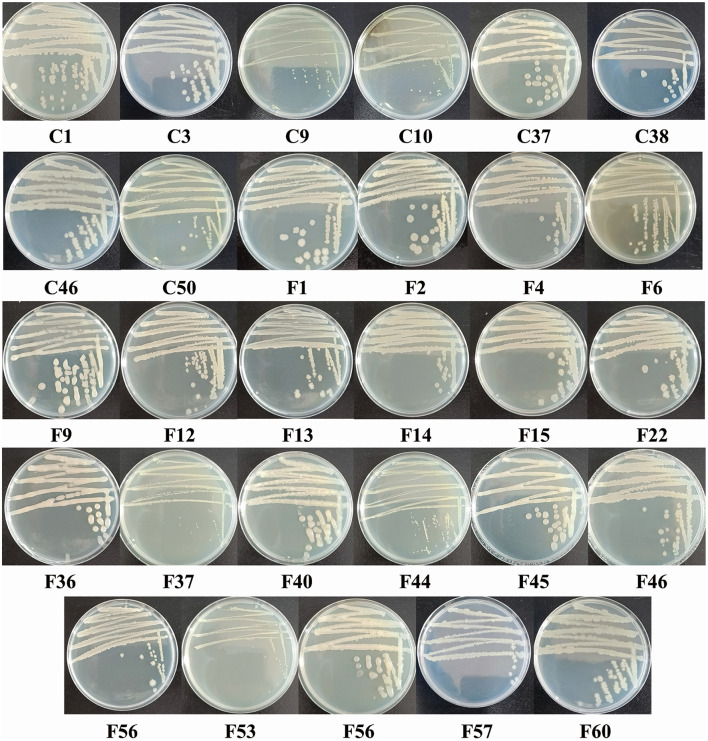
Colony morphology of endophytic bacterial isolates isolated from the pedicels of *C. reticulata* with antagonistic activity against nectar yeasts.

The 29 screened isolates of endophytic bacteria exhibited various morphological characteristics, such as distinct colors (including white, gray-white, and light yellow), margins (irregular, undulate, and entire), and shapes (rod and cocci) of colony (Figure 2). Gram staining results revealed that four isolates were Gram-negative, while the remaining isolates were all Gram-positive. All 29 isolates were positive for H_2_O_2_ and glucose metabolism. Nearly all isolates exhibited motility (except C18) and nitrate reductase activity (except for C10). Additionally, most isolates showed positive reactions for gelatin liquefaction (79.31%), methyl red (86.21%), and starch hydrolysis (86.21%). In contrast, only a few isolates showed positive responses for indole production (6.90%), V-P test (20.69%), and H_2_S production (20.69%) (Table 3).

**Table 3:** Characterization of endophytic bacteria isolated from the pedicels of *C. reticulata* with antagonistic activity against nectar yeasts.

**Isolates**	**Color**	**Margin**	**Shape**	**Gram staining**	**Motility**	**H_2_O_2_**	**Gelatin liquefaction**	**Indole production**	**Methyl red**	**V-P**	**H_2_S**	**Glucose**	**Nitrate reductase**	**Starch hydrolysis**
C1	Grayish white	Irregular	Rod	G+ve	+	+	+	–	+	–	–	+	+	+
C3	Creamy white	Undulate	Rod	G+ve	+	+	–	–	–	–	–	+	+	+
C9	Grayish white	Entire	Cocci	G–ve	+	+	+	–		–	–	+	+	+
C10	Grayish white	Entire	Rod	G+ve	+	+	+	–	+	–	–	+	–	+
C18	Yellowish white	Entire	Cocci	G+ve	–	+	–	–	+	+	–	+	+	–
C37	Creamy white	Undulate	Rod	G+ve	+	+	–	–	–	–	–	+	+	+
C38	Creamy white	Undulate	Rod	G+ve	+	+	–	–	–	–	–	+	+	+
C46	Grayish white	Undulate	Rod	G+ve	+	+	+	–	+	–	+	+	+	+
C50	White	Entire	Rod	G–ve	+	+	+	+	+	+	–	+	+	–
F1	White	Undulate	Rod	G+ve	+	+	+	–	+	–	–	+	+	+
F2	White	Undulate	Rod	G+ve	+	+	+	–	+	–	–	+	+	+
F4	Grayish white	Undulate	Rod	G+ve	+	+	+	–	+	+	+	+	+	+
F6	Creamy white	Entire	Cocci	G–ve	+	+	–	+	+	+	–	+	+	–
F9	White	Undulate	Rod	G+ve	+	+	+	–	+	–	–	+	+	+
F12	Grayish white	Undulate	Rod	G+ve	+	+	+	–	+	+	+	+	+	+
F13	Yellowish white	Undulate	Rod	G+ve	+	+	+	-	+	–	+	+	+	+
F14	Yellowish white	Undulate	Rod	G+ve	+	+	+	–	+	–	+	+	+	+
F15	White	Undulate	Rod	G+ve	+	+	+	–	+	–	–	+	+	+
F22	White	Undulate	Rod	G+ve	+	+	+	–	+	–	–	+	+	+
F36	White	Undulate	Rod	G+ve	+	+	+	–	+	–	–	+	+	+
F37	White	Undulate	Rod	G+ve	+	+	+	–	+	–	–	+	+	+
F40	White	Undulate	Rod	G+ve	+	+	+	–	+	–	–	+	+	+
F44	White	Undulate	Rod	G+ve	+	+	+	–	+	–	–	+	+	+
F45	White	Undulate	Rod	G+ve	+	+	+	–	+	–	–	+	+	+
F46	White	Undulate	Rod	G+ve	+	+	+	–	+	–	–	+	+	+
F50	White	Undulate	Rod	G+ve	+	+	+	–	+	–	–	+	+	+
F53	Yellow	Entire	Rod	G–ve	+	+	–	–	+	+	–	+	+	–
F56	Yellowish white	Undulate	Rod	G+ve	+	+	+	–	+	–	+	+	+	+
F60	White	Undulate	Rod	G+ve	+	+	+	–	+	–	–	+	+	+

“+” indicates positive, and “–” indicates negative. “G–ve” indicates Gram-negative bacteria, while “G+ve” indicates Gram-positive bacteria.

### 3.4 Identification of screened endophytic bacterial isolates

The 16S rRNA gene sequences were compared to known sequences in the GenBank database using BLAST. The results shown in Table 4 indicated 97-100% homology between the 16S rRNA sequences of 29 endophytic bacterial isolates and the known sequences. Among the 29 isolates, 23 belonged to the genus *Bacillus*, which accounted for the highest proportion. Specifically, the isolates were identified as *B. subtilis* (13 isolates), *B*. sp. (four isolates), *B. velezensis* (three isolates), *B. licheniformis* (two isolates), and *B. spizizenii* (one isolate). The remaining six isolates belonged to genus *Erwinia* (*E. billingiae*), *Pseudomonas* (*P. fluorescens* and *P. tolaasii*), *Cytobacillus* (*C. firmus*), *Staphylococcus* (*S. haemolyticus*), and *Pantoea* (*P. agglomerans*).

**Table 4:** Identification of endophytic bacterial isolates isolated from pedicels of *C. reticulata* with antagonistic activity against nectar yeasts.

**Isolates**	**Species**	**Length (bp)**	**Accession number**	**Closest number**	**Identity (%)**
C1	*Bacillus spizizenii*	1,453	PQ425620	MN443608.1	99.86%
C3	*Bacillus velezensis*	1,461	PQ425621	MT634570.1	99.93%
C9	*Pseudomonas fluorescens*	1,495	PQ425622	MW433874.1	99.38%
C10	*Cytobacillus firmus*	1,447	PQ425623	OQ568322.1	99.72%
C18	*Staphylococcus haemolyticus*	1,457	PQ425624	MG049771.1	99.72%
C37	*Bacillus velezensis*	1,457	PQ425625	MT634570.1	99.93%
C38	*Bacillus velezensis*	1,454	PQ425626	OP435752.1	99.79%
C46	*Bacillus sp*.	1,456	PQ425627	OP268582.1	100%
C50	*Erwinia billingiae*	1,427	PQ425628	OP102647.1	99.79%
F1	*Bacillus subtilis*	1,466	PQ425629	EU256502.1	98.22%
F2	*Bacillus subtilis*	1,460	PQ425630	OP753634.1	98.08%
F4	*Bacillus licheniformis*	1,461	PQ425631	ON597434.1	98.63%
F6	*Pseudomonas tolaasii*	1,443	PQ425632	MW326075.1	98.89%
F9	*Bacillus subtilis*	1,458	PQ425633	ON614206.1	98.35%
F12	*Bacillus licheniformis*	1,461	PQ425634	MW380593.1	98.01%
F13	*Bacillus sp*.	1,459	PQ425635	KJ955375.1	98.07%
F14	*Bacillus sp*.	1,475	PQ425636	MZ895421.1	97.90%
F15	*Bacillus subtilis*	1,463	PQ425637	OM362941.1	97.94%
F22	*Bacillus subtilis*	1,458	PQ425638	KC441785.1	98%
F36	*Bacillus subtilis*	1,460	PQ425639	ON584561.1	98.00%
F37	*Bacillus subtilis*	1,449	PQ425640	MZ895413.1	100%
F40	*Bacillus subtilis*	1,450	PQ425641	OQ165182.1	100%
F44	*Bacillus subtilis*	1,448	PQ425642	MT214144.1	100%
F45	*Bacillus subtilis*	1,448	PQ425643	MT111002.1	100%
F46	*Bacillus subtilis*	1,448	PQ425644	OP703559.1	100%
F50	*Bacillus subtilis*	1,468	PQ425645	MK823778.1	97.74%
F53	*Pantoea agglomerans*	1,437	PQ425646	MK883113.1	99.65%
F56	*Bacillus sp*.	1,470	PQ425647	MZ895421.1	97.88
F60	*Bacillus subtilis*	1,459	PQ425648	MZ895419.1	98.08%

### 3.5 Antagonistic effects of metabolites of *B. spizizenii* against nectar yeasts

The metabolites derived from *B. spizizenii* displayed potent antagonistic effects against *M. reukaufii, C. laurentii*, and *R. glutinis*, with MIC values of 62.5, 250, and 125 μg/mL, respectively. Notably, its antagonistic activity against these yeasts was comparable to that of the clinical drug amphotericin B (MIC values of 1.25, 0.63, and 1.25 μg/mL, respectively). These findings highlighted the significant potential of endophytic bacterium *B. spizizenii* to inhibit nectar yeasts.

### 3.6 Compositional analysis of the metabolites of *B. spizizenii*

**Figure 3:**
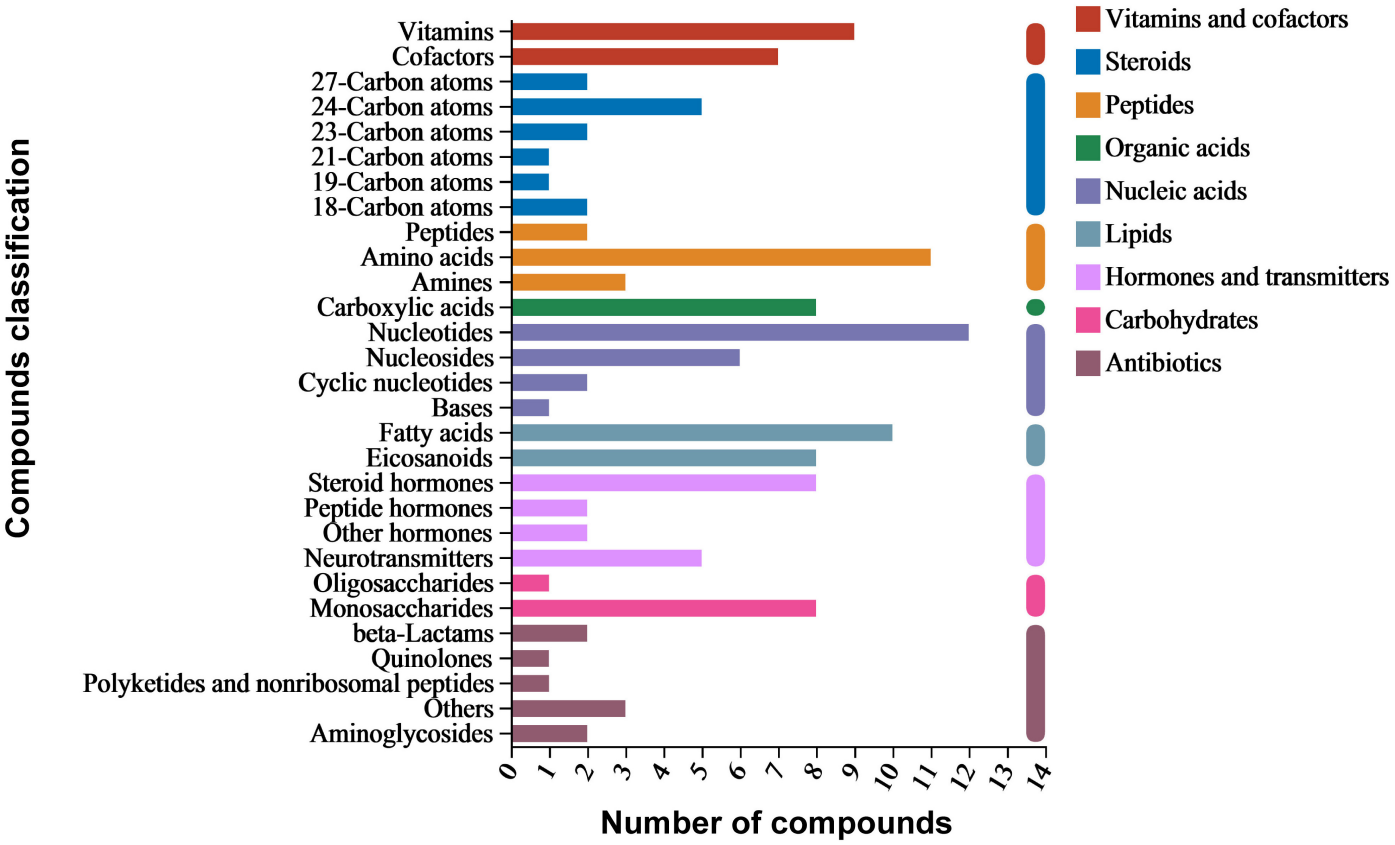
Compositional analysis of metabolites of *B. spizizenii* using KEGG database annotation.

Around 3,563 substances were detected in the metabolites of *B. spizizenii* using LC-MS/MS. KEGG enrichment analysis revealed nine categories of enriched substances: vitamins and cofactors, steroids, peptides, organic acids, nucleic acids, lipids, hormones and transmitters, carbohydrates, and antibiotics. Specifically, nucleotides accounted for the highest proportion of total compounds, followed by amino acids, fatty acids, vitamins, and monosaccharides. Antibiotic families, such as aminoglycosides, beta-lactams, and quinolones were also detected (Figure 3).

Active substances related to anti-yeast activity were detected in negative ion mode, including chloramphenicol, penicillin G, and spectinomycin. On the other hand, cephalosporin C, cycloheximide, griseofulvin, mitomycin, nalidixic acid, and streptomycin were detected in positive ion mode (Table 5). These antibiotics mainly belong to beta-lactams, polyketides and non-ribosomal peptides, quinolones, aminoglycosides, and other protein families. The results suggested that *B. spizizenii* inhibited the growth of nectar yeasts by producing multiple antibiotics, which indicates its great potential as a biocontrol bacterium.

**Table 5:** Major antibiotics present in the metabolites of *B. spizizenii*.

**ID**	**Metabolite**	**Category**	**Formula**
Pos_704	Cephalosporin C	beta-Lactams	C_16_H_21_N_3_O_8_S
neg_1293	Penicillin G	beta-Lactams	C_16_H_18_N_2_O_4_S
neg_1419	Spectinomycin	Aminoglycosides	C_14_H_24_N_2_O_7_
pos_1855	Streptomycin	Aminoglycosides	C_21_H_39_N_7_O_12_
pos_1056	Griseofulvin	Polyketides and non-ribosomal peptides	C_17_H_17_ClO_6_
pos_1412	Nalidixic Acid	Quinolones	C_12_H_12_N_2_O_3_
neg_495	Chloramphenicol	Others	C_11_H_12_C_l2_N_2_O_5_
Pos_776	Cycloheximide	Others	C_15_H_23_NO_4_
pos_1316	Mitomycin	Others	C_15_H_18_N_4_O_5_

**Figure 4:**
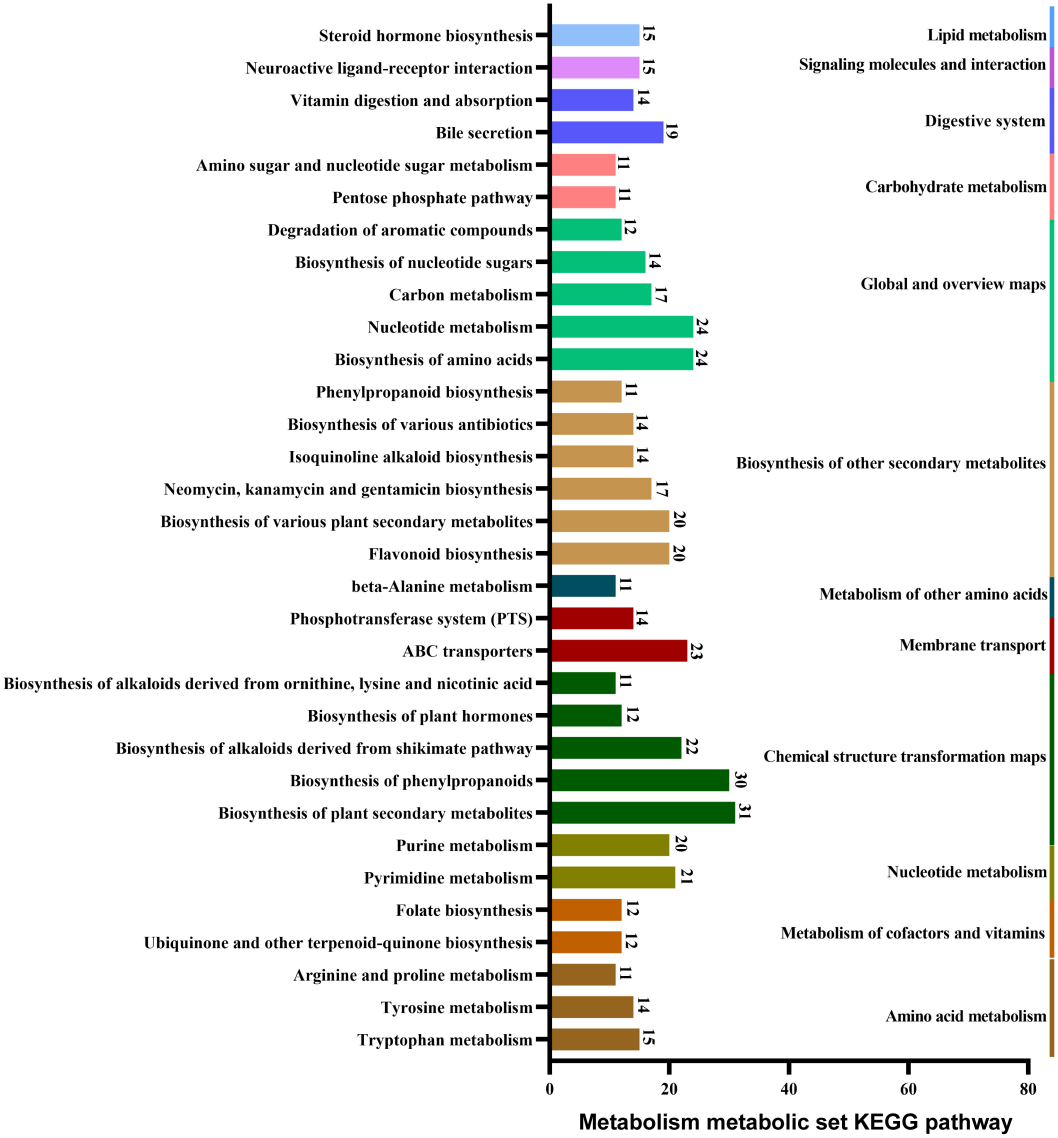
Major pathways involved in the metabolism of *B. spizizenii*, revealed by KEGG database annotations.

KEGG database annotations revealed the major pathways involved in the production of metabolites in *B. spizizenii*. These pathways primarily included the biosynthesis of plant secondary metabolites, phenylpropanoid, and amino acid, as well as nucleotide metabolism, ABC transportation, and pyrimidine metabolism (Figure 4).

## 4 Discussion

Endophytic bacteria are commonly present in plants. As research continues to expand, endophytic bacteria have been increasingly isolated and identified from plant tissues (Hardoim et al., 2015). However, *C. reticulata* has been rarely explored, especially in its native Yunnan Province. Using surface sterilization, 138 endophytic bacterial isolates were isolated from the pedicels of 12- and 60-year-old *C. reticulata* trees. The density and diversity of endophytic bacterial isolates in the pedicels of 60-year-old *C. reticulata* were higher than those of the 12-year-old pedicel tissues. This finding was consistent with the results obtained through high-throughput sequencing (Huang et al., 2023). The invasion and colonization of host plant by endophytic bacteria represent dynamic processes in terms of time and space. Endophytic bacteria not only modify the colonization sites through transpiration, but also exhibit temporal changes in the extent of colonization and species diversity. As plants grow, density and diversity of endophytic bacteria increase (Wagner et al., 2016; Liu et al., 2017). Throughout the lifespan of plants, tissues with longer inoculation periods tend to exhibit higher richness of endophytic bacteria, which may be attributed to larger colonization amount and longer microbial growth period (Ercolani, 1991). In old trees, richness and diversity of endophytic bacteria are significantly higher due to long-term accumulation and co-evolutionary processes. These trees serve as natural microbial resource libraries, providing valuable resources for screening, investigating, and utilizing the endophytic bacteria living within plants.

Using beneficial microorganisms and their metabolites is an effective biocontrol method to prevent and control diseases in agricultural and forest crops (Abbasi et al., 2002). The biocontrol mechanisms of endophytic bacteria primarily involve: (1) competition for advantageous ecological niches and nutrients against pathogens, (2) the secretion of antimicrobial substances that provide antibacterial activity, (3) parasitism on pathogens, (4) the induction of systemic resistance in host plants to pathogens, and (5) the regulation of the microecological system of the host plant (Compant et al., 2005; Ansari and Mahmood, 2019; Kumar and Singh, 2020). Ultimately, these mechanisms promote host plant growth or enhance resistance, thereby enabling effective defense against plant pathogens. Thus, endophytic bacteria help host plants respond to microbial infestation, suggesting that these bacteria may also assist plant nectar in antagonizing nectar yeast. Due to the nectar is nutritious, it attracts colonization by yeasts. However, the active metabolism of these yeasts depletes the nutritional components of the nectar, significantly reducing the quality of the rewards for pollinators and ultimately weakening the reproductive fitness of cross-pollinated plants (Herrera et al., 2008; Canto and Herrera, 2012). Therefore, it was essential to isolate numerous yeast-inhibiting endophytic bacterial isolates from the pedicels of *C. reticulata* to study the role of endophytes in maintaining the stability and quality of nectar.

The endophytic bacteria isolated from the pedicel of *C. reticulata* were diverse. In the present study, 29 endophytic bacterial isolates exhibited inhibitory effects against at least one species of nectar yeast. 16S rRNA sequence analysis revealed that the isolates belonged to six different genera, including *Bacillus, Erwinia, Pseudomonas, Cytobacillus, Staphylococcus*, and *Pantoea*, with most isolates belonging to genus *Bacillus*. Endophytic bacteria belonging to the genera *Bacillus, Pseudomonas, Enterobacter* and *Sporosarcina* have been widely reported previously in many plants (Li et al., 2012; Cabanás et al., 2014). *Bacillus* are Gram-positive bacteria that have become a biocontrol research hotspot, as they can produce a variety of antimicrobial substances (Ongena and Jacques, 2008; Sumi et al., 2015). The metabolites of *Bacillus* include glucanase, protease, cellulase, chitinase, etc., which inhibit the growth of pathogens by degrading their cell walls. They can also produce lipopeptide substances that resist the invasion and harmful effects of plant pathogens. Furthermore, they promote plant growth by competing with pathogenic bacteria for nutrients, secreting antimicrobial peptides and antibiotics, and inducing the systemic disease resistance in plants (Pan et al., 2017). In this study, some endophytic bacteria of genus *Bacillus* showed at least one or more enzyme activities and could utilize various substrates, such as glucose, nitrate, and starch. This finding suggests that these bacteria can promote the utilization of energy in the host plant.

The fermentation substances of isolate C1, identified as *B. spizizenii*, exhibited the best antagonistic activity against yeasts. This antagonistic activity was comparable to the activity shown by positive control, i.e., amphotericin B. Furthermore, *B. spizizenii* could produce diverse antibiotics belonging to aminoglycosides, Macrolides, and quinolones classes, such as spectinomycin, bacillomycin D, and macrolactin (Chen et al., 2021; Han et al., 2021). Previous studies have also shown the ability of *B. spizizenii* to secrete the antibiotics of other classes, as well as several lipophilic membrane-active agents, such as subtilin, subtilosin, surfactin, and fengycin (Kim et al., 2017; Lim et al., 2017; Zhao et al., 2018). These findings suggest that the antagonistic activity of *B. spizizenii* against nectar yeasts can be attributed to its antibiotic-secreting properties. The main metabolic pathways of *B. spizizenii* included the biosynthesis of plant secondary metabolites, phenylpropanoid compounds, amino acids, nucleotide metabolism, and other compounds. Additionally, other metabolic pathways were also discovered, such as the biosynthesis of neomycin, kanamycin, kanosamine, etc. These metabolites and pathways provide new research directions to further explore the antimicrobial mechanism of *B. spizizenii*. *Camellia reticulata* secretes a large amount of nectar and relies on pollinators for reproduction. The antimicrobial substances in the nectar are generally believed to be derived from the active substances produced by the plant itself. In addition, the endophytic bacteria colonizing the pedicels also produce abundant secondary metabolites and antibiotics, which may be crucial for maintaining the nectar homeostasis. This finding may provide new ideas for the analysis of response mechanisms of heterologous pollinating plants to microbial infestation. However, the relevant conclusions need to be verified by further research. Exploring the potential of these metabolites and antibiotics in biological control and understanding the underlying mechanisms is also an important direction for future research.

## 5 Conclusion

The pedicels of *C. reticulata* are rich in endophytic bacteria. In this study, 29 yeast-resisting endophytic bacteria were isolated and screened using spot inoculation and oxford cup methods. Most of the isolates belonged to *Bacillus* genus, with a few isolates belonging to *Erwinia, Pseudomonas, Cytobacillus, Staphylococcus*, and *Pantoea*. Especially, the endophytic bacterium C1, identified as *B. spizizenii*, exhibited the strongest inhibitory effect on nectar yeasts. This isolate was found to be a source of structurally and functionally diverse natural compounds, exhibiting anti-yeast effects through the synthesis of antibiotics, such as chloramphenicol, penicillin G, spectinomycin, etc. This study highlighted the potential uses of endophytic bacteria in maintaining nectar quality and stability, as well as the reproductive fitness of host plant, providing new insights into the role of endophytic bacteria of pedicels in nectar resistance against yeasts.

## Data Availability

The datasets presented in this study can be found in online repositories. The names of the repository/repositories and accession number(s) can be found in the article/Supplementary material.
