# Supplementary material for: Endophytic bacteria in Camellia reticulata pedicels: isolation, screening and analysis of antagonistic activity against nectar yeasts
Source: Front Microbiol. 2024 Oct 21;15:1459354. doi: 10.3389/fmicb.2024.1459354 (PMC11533746; doi:10.3389/fmicb.2024.1459354)
Supplement: Supplementary file 1 [file Data_Sheet_1.docx]

Supplementary Material

# Supplementary Table

**Supplementary Table 1.** The isolates of endophytic bacteria from *Camellia reticulata* pedicels and their GenBank accession numbers

| Isolates | Accession | Release Date |
| --- | --- | --- |
| C1 | PQ425620 | 10/09/2024 |
| C3 | PQ425621 | 10/09/2024 |
| C9 | PQ425622 | 10/09/2024 |
| C10 | PQ425623 | 10/09/2024 |
| C18 | PQ425624 | 10/09/2024 |
| C37 | PQ425625 | 10/09/2024 |
| C38 | PQ425626 | 10/09/2024 |
| C46 | PQ425627 | 10/09/2024 |
| C50 | PQ425628 | 10/09/2024 |
| F1 | PQ425629 | 10/09/2024 |
| F2 | PQ425630 | 10/09/2024 |
| F4 | PQ425631 | 10/09/2024 |
| F6 | PQ425632 | 10/09/2024 |
| F9 | PQ425633 | 10/09/2024 |
| F12 | PQ425634 | 10/09/2024 |
| F13 | PQ425635 | 10/09/2024 |
| F14 | PQ425636 | 10/09/2024 |
| F15 | PQ425637 | 10/09/2024 |
| F22 | PQ425638 | 10/09/2024 |
| F36 | PQ425639 | 10/09/2024 |
| F37 | PQ425640 | 10/09/2024 |
| F40s | PQ425641 | 10/09/2024 |
| F44 | PQ425642 | 10/09/2024 |
| F45 | PQ425643 | 10/09/2024 |
| F46 | PQ425644 | 10/09/2024 |
| F50 | PQ425645 | 10/09/2024 |
| F53 | PQ425646 | 10/09/2024 |
| F56 | PQ425647 | 10/09/2024 |
| F60 | PQ425648 | 10/09/2024 |

# Supplementary Data

**This is derived from the data uploaded to GenBank**

LOCUS PQ425620 1460 bp DNA linear BCT 04-OCT-2024

DEFINITION Bacillus spizizenii strain C1 16S ribosomal RNA gene, partial sequence.

ACCESSION PQ425620

VERSION PQ425620

KEYWORDS .

SOURCE Bacillus spizizenii

ORGANISM Bacillus spizizenii

Bacteria; Bacillota; Bacilli; Bacillales; Bacillaceae; Bacillus.

REFERENCE 1 (bases 1 to 1460)

AUTHORS Huang,R.

TITLE Direct Submission

JOURNAL Submitted (04-OCT-2024) College of Animal Science and Technology,

Yunnan Agricultural University, Fengyuan Road No. 452, Kunming,

Yunnan 650201, China

COMMENT ##Assembly-Data-START##

Sequencing Technology :: Sanger dideoxy sequencing

##Assembly-Data-END##

FEATURES Location/Qualifiers

source 1..1460

/organism="Bacillus spizizenii"

/mol_type="genomic DNA"

/strain="C1"

/db_xref="taxon:96241"

/geo_loc_name="China: Yunnan, Tengchong County"

/collection_date="May-2023"

rRNA <1..>1460

/product="16S ribosomal RNA"

ORIGIN

1 cgggaatggc ggcgtgccta tacatgcagt cgagcggaca gatgggagct tgctccctga

61 tgttagcggc ggacgggtga gtaacacgtg ggtaacctgc ctgtaagact gggataactc

121 cgggaaaccg gggctaatac cggatggttg tttgaaccgc atggttcaaa cataaaaggt

181 ggcttcggct accacttaca gatggacccg cggcgcatta gctagttggt gaggtaacgg

241 ctcaccaagg caacgatgcg tagccgacct gagagggtga tcggccacac tgggactgag

301 acacggccca gactcctacg ggaggcagca gtagggaatc ttccgcaatg gacgaaagtc

361 tgacggagca acgccgcgtg agtgatgaag gttttcggat cgtaaagctc tgttgttagg

421 gaagaacaag taccgttcga atagggcggt accttgacgg tacctaacca gaaagccacg

481 gctaactacg tgccagcagc cgcggtaata cgtaggtggc aagcgttgtc cggaattatt

541 gggcgtaaag ggctcgcagg cggtttctta agtctgatgt gaaagccccc ggctcaaccg

601 gggagggtca ttggaaactg gggaacttga gtgcagaaga ggagagtgga attccacgtg

661 tagcggtgaa atgcgtagag atgtggagga acaccagtgg cgaaggcgac tctctggtct

721 gtaactgacg ctgaggagcg aaagcgtggg gagcgaacag gattagatac cctggtagtc

781 cacgccgtaa acgatgagtg ctaagtgtta gggggtttcc gccccttagt gctgcagcta

841 acgcattaag cactccgcct ggggagtacg gtcgcaagac tgaaactcaa aggaattgac

901 gggggcccgc acaagcggtg gagcatgtgg tttaattcga agcaacgcga agaaccttac

961 caggtcttga catcctctga caatcctaga gataggacgt ccccttcggg ggcagagtga

1021 caggtggtgc atggttgtcg tcagctcgtg tcgtgagatg ttgggttaag tcccgcaacg

1081 agcgcaaccc ttgatcttag ttgccagcat tcagttgggc actctaaggt gactgccggt

1141 gacaaaccgg aggaaggtgg ggatgacgtc aaatcatcat gccccttatg acctgggcta

1201 cacacgtgct acaatggaca gaacaaaggg cagcgaaacc gcgaggttaa gccaatccca

1261 caaatctgtt ctcagttcgg atcgcagtct gcaactcgac tgcgtgaagc tggaatcgct

1321 agtaatcgcg gatcagcatg ccgcggtgaa tacgttcccg ggccttgtac acaccgcccg

1381 tcacaccacg agagtttgta acacccgaag tcggtgaggt aaccttttag gagccagccg

1441 ccgaatggtg acaagaggtt

//

LOCUS PQ425621 1460 bp DNA linear BCT 04-OCT-2024

DEFINITION Bacillus velezensis strain C3 16S ribosomal RNA gene, partial sequence.

ACCESSION PQ425621

VERSION PQ425621

KEYWORDS .

SOURCE Bacillus velezensis

ORGANISM Bacillus velezensis

Bacteria; Bacillota; Bacilli; Bacillales; Bacillaceae; Bacillus;

Bacillus amyloliquefaciens group.

REFERENCE 1 (bases 1 to 1460)

AUTHORS Huang,R.

TITLE Direct Submission

JOURNAL Submitted (04-OCT-2024) College of Animal Science and Technology,

Yunnan Agricultural University, Fengyuan Road No. 452, Kunming,

Yunnan 650201, China

COMMENT ##Assembly-Data-START##

Sequencing Technology :: Sanger dideoxy sequencing

##Assembly-Data-END##

FEATURES Location/Qualifiers

source 1..1460

/organism="Bacillus velezensis"

/mol_type="genomic DNA"

/strain="C3"

/db_xref="taxon:492670"

/geo_loc_name="China: Yunnan, Tengchong County"

/collection_date="May-2023"

rRNA <1..>1460

/product="16S ribosomal RNA"

ORIGIN

1 cggggattgg cggcgtgcta tacatgcaag tcgagcggac agatgggagc ttgctccctg

61 atgttagcgg cggacgggtg agtaacacgt gggtaacctg cctgtaagac tgggataact

121 ccgggaaacc ggggctaata ccggatggtt gtttgaaccg catggttcaa acataaaagg

181 tggcttcggc taccacttac agatggaccc gcggcgcatt agctagttgg tgaggtaacg

241 gctcaccaag gcaacgatgc gtagccgacc tgagagggtg atcggccaca ctgggactga

301 gacacggccc agactcctac gggaggcagc agtagggaat cttccgcaat ggacgaaagt

361 ctgacggagc aacgccgcgt gagtgatgaa ggttttcgga tcgtaaagct ctgttgttag

421 ggaagaacaa gtaccgttcg aatagggcgg taccttgacg gtacctaacc agaaagccac

481 ggctaactac gtgccagcag ccgcggtaat acgtaggtgg caagcgttgt ccggaattat

541 tgggcgtaaa gggctcgcag gcggtttctt aagtctgatg tgaaagcccc cggctcaacc

601 ggggagggtc attggaaact ggggaacttg agtgcagaag aggagagtgg aattccacgt

661 gtagcggtga aatgcgtaga gatgtggagg aacaccagtg gcgaaggcga ctctctggtc

721 tgtaactgac gctgaggagc gaaagcgtgg ggagcgaaca ggattagata ccctggtagt

781 ccacgccgta aacgatgagt gctaagtgtt agggggtttc cgccccttag tgctgcagct

841 aacgcattaa gcactccgcc tggggagtac ggtcgcaaga ctgaaactca aaggaattga

901 cgggggcccg cacaagcggt ggagcatgtg gtttaattcg aagcaacgcg aagaacctta

961 ccaggtcttg acatcctctg acaatcctag agataggacg tccccttcgg gggcagagtg

1021 acaggtggtg catggttgtc gtcagctcgt gtcgtgagat gttgggttaa gtcccgcaac

1081 gagcgcaacc cttgatctta gttgccagca ttcagttggg cactctaagg tgactgccgg

1141 tgacaaaccg gaggaaggtg gggatgacgt caaatcatca tgccccttat gacctgggct

1201 acacacgtgc tacaatggac agaacaaagg gcagcgaaac cgcgaggtta agccaatccc

1261 acaaatctgt tctcagttcg gatcgcagtc tgcaactcga ctgcgtgaag ctggaatcgc

1321 tagtaatcgc ggatcagcat gccgcggtga atacgttccc gggccttgta cacaccgccc

1381 gtcacaccac gagagtttgt aacacccgaa gtcggtgagg taacctttta ggagccagcc

1441 gccgaaggtg aacagaattg

//

LOCUS PQ425622 1451 bp DNA linear BCT 04-OCT-2024

DEFINITION Pseudomonas fluorescens strain C9 16S ribosomal RNA gene, partial sequence.

ACCESSION PQ425622

VERSION PQ425622

KEYWORDS .

SOURCE Pseudomonas fluorescens

ORGANISM Pseudomonas fluorescens

Bacteria; Pseudomonadota; Gammaproteobacteria; Pseudomonadales;

Pseudomonadaceae; Pseudomonas.

REFERENCE 1 (bases 1 to 1451)

AUTHORS Huang,R.

TITLE Direct Submission

JOURNAL Submitted (04-OCT-2024) College of Animal Science and Technology,

Yunnan Agricultural University, Fengyuan Road No. 452, Kunming,

Yunnan 650201, China

COMMENT ##Assembly-Data-START##

Sequencing Technology :: Sanger dideoxy sequencing

##Assembly-Data-END##

FEATURES Location/Qualifiers

source 1..1451

/organism="Pseudomonas fluorescens"

/mol_type="genomic DNA"

/strain="C9"

/db_xref="taxon:294"

/geo_loc_name="China: Yunnan, Tengchong County"

/collection_date="May-2023"

rRNA <1..>1451

/product="16S ribosomal RNA"

ORIGIN

1 cacgcgttgg ccggcatgtc taacacatag cagtcgagcg gtagagagaa gcttgcttct

61 cttgagagcg gcggacgggt gagtaatgcc taggaatctg cctggtagtg ggggataacg

121 ttcggaaacg gacgctaata ccgcatacgt cctacgggag aaagcagggg accttcgggc

181 cttgcgctat cagatgagcc taggtcggat tagctagttg gtgaggtaat ggctcaccaa

241 ggcgacgatc cgtaactggt ctgagaggat gatcagtcac actggaactg agacacggtc

301 cagactccta cgggaggcag cagtggggaa tattggacaa tgggcgaaag cctgatccag

361 ccatgccgcg tgtgtgaaga aggtcttcgg attgtaaagc actttaagtt gggaggaagg

421 gcattaacct aatacgttag tgttttgacg ttaccgacag aataagcacc ggctaactct

481 gtgccagcag ccgcggtaat acagagggtg caagcgttaa tcggaattac tgggcgtaaa

541 gcgcgcgtag gtggtttgtt aagttggatg tgaaatcccc gggctcaacc tgggaactgc

601 attcaaaact gactgactag agtatggtag agggtggtgg aatttcctgt gtagcggtga

661 aatgcgtaga tataggaagg aacaccagtg gcgaaggcga ccacctggac taatactgac

721 actgaggtgc gaaagcgtgg ggagcaaaca ggattagata ccctggtagt ccacgccgta

781 aacgatgtca actagccgtt ggaagccttg agcttttagt ggcgcagcta acgcattaag

841 ttgaccgcct ggggagtacg gccgcaaggt taaaactcaa atgaattgac gggggcccgc

901 acaagcggtg gagcatgtgg tttaattcga agcaacgcga agaaccttac caggccttga

961 catccaatga actttctaga gatagattgg ttgccttcgg gaacattgag acaggtgctg

1021 catggctgtc gtcagctcgt gtcgtgagat gttgggttaa gtcccgtaac gagcgcaacc

1081 cttgtcctta gttaccagca cgtaatggtg ggcactctaa ggagactgcc ggtgacaaac

1141 cggaggaagg tggggatgac gtcaagtcat catggccctt acggcctggg ctacacacgt

1201 gctacaatgg tcggtacaga gggttgccaa gccgcgaggt ggagctaatc ccataaaacc

1261 gatcgtagtc cggatcgcag tctgcaactc gactgcgtga agtcggaatc gctagtaatc

1321 gcgaatcaga atgtcgcggt gaatacgttc ccgggccttg tacacaccgc ccgtcacacc

1381 atgggagtgg gttgcaccag aagtagctag tctaaccttc gggaggacgg taccacggtg

1441 tattactttg g

//

LOCUS PQ425623 1447 bp DNA linear BCT 04-OCT-2024

DEFINITION Cytobacillus firmus strain C10 16S ribosomal RNA gene, partial sequence.

ACCESSION PQ425623

VERSION PQ425623

KEYWORDS .

SOURCE Cytobacillus firmus

ORGANISM Cytobacillus firmus

Bacteria; Bacillota; Bacilli; Bacillales; Bacillaceae;

Cytobacillus.

REFERENCE 1 (bases 1 to 1447)

AUTHORS Huang,R.

TITLE Direct Submission

JOURNAL Submitted (04-OCT-2024) College of Animal Science and Technology,

Yunnan Agricultural University, Fengyuan Road No. 452, Kunming,

Yunnan 650201, China

COMMENT ##Assembly-Data-START##

Sequencing Technology :: Sanger dideoxy sequencing

##Assembly-Data-END##

FEATURES Location/Qualifiers

source 1..1447

/organism="Cytobacillus firmus"

/mol_type="genomic DNA"

/strain="C10"

/db_xref="taxon:1399"

/geo_loc_name="China: Yunnan, Tengchong County"

/collection_date="May-2023"

rRNA <1..>1447

/product="16S ribosomal RNA"

ORIGIN

1 cggggggtgc tatacatgca gtcgagcgga cggatgggag cttgctccca gaccgtcagc

61 ggcggacggg tgagtaacac gtgggcaacc tgcctgtaag actgggataa ctccgggaaa

121 ccggggctaa taccggataa ttcttttcct cacatgagga aaagctgaaa gatggcatct

181 cgctatcact tacagatggg cccgcggcgc attagctagt tggtgaggta acggctcacc

241 aaggcgacga tgcgtagccg acctgagagg gtgatcggcc acactgggac tgagacacgg

301 cccagactcc tacgggaggc agcagtaggg aatcttccgc aatggacgaa agtctgacgg

361 agcaacgccg cgtgagtgat gaaggttttc ggatcgtaaa actctgttgt cagggaagaa

421 caagtaccgg agtaactgcc ggtaccttga cggtacctga ccagaaagcc acggctaact

481 acgtgccagc agccgcggta atacgtaggt ggcaagcgtt gtccggaatt attgggcgta

541 aagcgcgcgc aggcggttcc ttaagtctga tgtgaaagcc cccggctcaa ccggggaggg

601 tcattggaaa ctggggaact tgagtgcaga agagaagagt ggaattccac gtgtagcggt

661 gaaatgcgta gagatgtgga ggaacaccag tggcgaaggc gactctttgg tctgtaactg

721 acgctgaggc gcgaaagcgt ggggagcaaa caggattaga taccctggta gtccacgccg

781 taaacgatga gtgctaagtg ttagagggtt tccgcccttt agtgctgcag caaacgcatt

841 aagcactccg cctggggagt acggccgcaa ggctgaaact caaaggaatt gacgggggcc

901 cgcacaagcg gtggagcatg tggtttaatt cgaagcaacg cgaagaacct taccaggtct

961 tgacatctcc tgacaaccct agagataggg cgttcccctt cgggggacag gatgacaggt

1021 ggtgcatggt tgtcgtcagc tcgtgtcgtg agatgttggg ttaagtcccg caacgagcgc

1081 aacccttgat cttagttgcc agcattcagt tgggcactct aaggtgactg ccggtgacaa

1141 accggaggaa ggtggggatg acgtcaaatc atcatgcccc ttatgacctg ggctacacac

1201 gtgctacaat ggatggtaca aagggctgca agaccgcgag gttaagcgaa tcccataaaa

1261 ccattctcag ttcggattgc aggctgcaac tcgcctgcat gaagccggaa tcgctagtaa

1321 tcgcggatca gcatgccgcg gtgaatacgt tcccgggcct tgtacacacc gcccgtcaca

1381 ccacgagagt ttgtaacacc cgaagtcggt ggggtaacct tttggagcca gccgctaagg

1441 tgatctg

//

LOCUS PQ425624 1464 bp DNA linear BCT 04-OCT-2024

DEFINITION Staphylococcus haemolyticus strain C18 16S ribosomal RNA gene, partial sequence.

ACCESSION PQ425624

VERSION PQ425624

KEYWORDS .

SOURCE Staphylococcus haemolyticus

ORGANISM Staphylococcus haemolyticus

Bacteria; Bacillota; Bacilli; Bacillales; Staphylococcaceae;

Staphylococcus.

REFERENCE 1 (bases 1 to 1464)

AUTHORS Huang,R.

TITLE Direct Submission

JOURNAL Submitted (04-OCT-2024) College of Animal Science and Technology,

Yunnan Agricultural University, Fengyuan Road No. 452, Kunming,

Yunnan 650201, China

COMMENT ##Assembly-Data-START##

Sequencing Technology :: Sanger dideoxy sequencing

##Assembly-Data-END##

FEATURES Location/Qualifiers

source 1..1464

/organism="Staphylococcus haemolyticus"

/mol_type="genomic DNA"

/strain="C18"

/db_xref="taxon:1283"

/geo_loc_name="China: Yunnan, Tengchong County"

/collection_date="May-2023"

rRNA <1..>1464

/product="16S ribosomal RNA"

ORIGIN

1 taggctctgt ggcggcgtgc tatacatgca agtcgagcga acggataagg agcttgctcc

61 tttgaagtta gcggcggacg ggtgagtaac acgtgggtaa cctacctata agactggaat

121 aacttcggga aaccggagct aatgccggat aacatttgga accgcatggt tctaaagtaa

181 aagatggttt tgctatcact tatagatgga cccgcgccgt attagctagt tggtaaggta

241 acggcttacc aaggcaacga tacgtagccg acctgagagg gtgatcggcc acactggaac

301 tgagacacgg tccagactcc tacgggaggc agcagtaggg aatcttccgc aatggacgaa

361 agtctgacgg agcaacgccg cgtgagtgat gaaggttttc ggatcgtaaa actctgttat

421 tagggaagaa caaatgtgta agtaactgtg cacatcttga cggtacctaa tcagaaagcc

481 acggctaact acgtgccagc agccgcggta atacgtaggt ggcaagcgtt atccggaatt

541 attgggcgta aagcgcgcgt aggcggtttc ttaagtctga tgtgaaagcc cacggctcaa

601 ccgtggaggg tcattggaaa ctgggaaact tgagtacaga agaggaaagt ggaattccat

661 gtgtagcggt gaaatgcgca gagatatgga ggaacaccag tggcgaaggc gactttctgg

721 tctgtaactg acgctgatgt gcgaaagcgt ggggatcaaa caggattaga taccctggta

781 gtccacgccg taaacgatga gtgctaagtg ttagggggtt tccgcccctt agtgctgcag

841 ctaacgcatt aagcactccg cctggggagt acgaccgcaa ggttgaaact caaaggaatt

901 gacggggacc cgcacaagcg gtggagcatg tggtttaatt cgaagcaacg cgaagaacct

961 taccaaatct tgacatcctt tgaaaactct agagatagag ctttcccctt cgggggacaa

1021 agtgacaggt ggtgcatggt tgtcgtcagc tcgtgtcgtg agatgttggg ttaagtcccg

1081 caacgagcgc aacccttaaa cttagttgcc agcatttagt tgggcactct aggttgactg

1141 ccggtgacaa accggaggaa ggtggggatg acgtcaaatc atcatgcccc ttatgatttg

1201 ggctacacac gtgctacaat ggacaataca aagggcagct aaaccgcgag gtcatgcaaa

1261 tcccataaag ttgttctcag ttcggattgt agtctgcaac tcgactacat gaagctggaa

1321 tcgctagtaa tcgtagatca gcatgctacg gtgaatacgt tcccgggtct tgtacacacc

1381 gcccgtcaca ccacgagagt ttgtaacacc cgaagccggt ggagtaacca tttatggagc

1441 tagccgtcga agtgaccaaa atgg

//

LOCUS PQ425625 1458 bp DNA linear BCT 04-OCT-2024

DEFINITION Bacillus velezensis strain C37 16S ribosomal RNA gene, partial sequence.

ACCESSION PQ425625

VERSION PQ425625

KEYWORDS .

SOURCE Bacillus velezensis

ORGANISM Bacillus velezensis

Bacteria; Bacillota; Bacilli; Bacillales; Bacillaceae; Bacillus;

Bacillus amyloliquefaciens group.

REFERENCE 1 (bases 1 to 1458)

AUTHORS Huang,R.

TITLE Direct Submission

JOURNAL Submitted (04-OCT-2024) College of Animal Science and Technology,

Yunnan Agricultural University, Fengyuan Road No. 452, Kunming,

Yunnan 650201, China

COMMENT ##Assembly-Data-START##

Sequencing Technology :: Sanger dideoxy sequencing

##Assembly-Data-END##

FEATURES Location/Qualifiers

source 1..1458

/organism="Bacillus velezensis"

/mol_type="genomic DNA"

/strain="C37"

/db_xref="taxon:492670"

/geo_loc_name="China: Yunnan, Tengchong County"

/collection_date="May-2023"

rRNA <1..>1458

/product="16S ribosomal RNA"

ORIGIN

1 gtggatggcg gcgtgctata catgcaagtc gagcggacag atgggagctt gctccctgat

61 gttagcggcg gacgggtgag taacacgtgg gtaacctgcc tgtaagactg ggataactcc

121 gggaaaccgg ggctaatacc ggatggttgt ttgaaccgca tggttcaaac ataaaaggtg

181 gcttcggcta ccacttacag atggacccgc ggcgcattag ctagttggtg aggtaacggc

241 tcaccaaggc aacgatgcgt agccgacctg agagggtgat cggccacact gggactgaga

301 cacggcccag actcctacgg gaggcagcag tagggaatct tccgcaatgg acgaaagtct

361 gacggagcaa cgccgcgtga gtgatgaagg ttttcggatc gtaaagctct gttgttaggg

421 aagaacaagt accgttcgaa tagggcggta ccttgacggt acctaaccag aaagccacgg

481 ctaactacgt gccagcagcc gcggtaatac gtaggtggca agcgttgtcc ggaattattg

541 ggcgtaaagg gctcgcaggc ggtttcttaa gtctgatgtg aaagcccccg gctcaaccgg

601 ggagggtcat tggaaactgg ggaacttgag tgcagaagag gagagtggaa ttccacgtgt

661 agcggtgaaa tgcgtagaga tgtggaggaa caccagtggc gaaggcgact ctctggtctg

721 taactgacgc tgaggagcga aagcgtgggg agcgaacagg attagatacc ctggtagtcc

781 acgccgtaaa cgatgagtgc taagtgttag ggggtttccg ccccttagtg ctgcagctaa

841 cgcattaagc actccgcctg gggagtacgg tcgcaagact gaaactcaaa ggaattgacg

901 ggggcccgca caagcggtgg agcatgtggt ttaattcgaa gcaacgcgaa gaaccttacc

961 aggtcttgac atcctctgac aatcctagag ataggacgtc cccttcgggg gcagagtgac

1021 aggtggtgca tggttgtcgt cagctcgtgt cgtgagatgt tgggttaagt cccgcaacga

1081 gcgcaaccct tgatcttagt tgccagcatt cagttgggca ctctaaggtg actgccggtg

1141 acaaaccgga ggaaggtggg gatgacgtca aatcatcatg ccccttatga cctgggctac

1201 acacgtgcta caatggacag aacaaagggc agcgaaaccg cgaggttaag ccaatcccac

1261 aaatctgttc tcagttcgga tcgcagtctg caactcgact gcgtgaagct ggaatcgcta

1321 gtaatcgcgg atcagcatgc cgcggtgaat acgttcccgg gccttgtaca caccgcccgt

1381 cacaccacga gagtttgtaa cacccgaagt cggtgaggta accttttagg agccagccgc

1441 cgaaggtgaa cagaaatt

//

LOCUS PQ425626 1460 bp DNA linear BCT 04-OCT-2024

DEFINITION Bacillus velezensis strain C38 16S ribosomal RNA gene, partial sequence.

ACCESSION PQ425626

VERSION PQ425626

KEYWORDS .

SOURCE Bacillus velezensis

ORGANISM Bacillus velezensis

Bacteria; Bacillota; Bacilli; Bacillales; Bacillaceae; Bacillus;

Bacillus amyloliquefaciens group.

REFERENCE 1 (bases 1 to 1460)

AUTHORS Huang,R.

TITLE Direct Submission

JOURNAL Submitted (04-OCT-2024) College of Animal Science and Technology,

Yunnan Agricultural University, Fengyuan Road No. 452, Kunming,

Yunnan 650201, China

COMMENT ##Assembly-Data-START##

Sequencing Technology :: Sanger dideoxy sequencing

##Assembly-Data-END##

FEATURES Location/Qualifiers

source 1..1460

/organism="Bacillus velezensis"

/mol_type="genomic DNA"

/strain="C38"

/db_xref="taxon:492670"

/geo_loc_name="China: Yunnan, Tengchong County"

/collection_date="May-2023"

rRNA <1..>1460

/product="16S ribosomal RNA"

ORIGIN

1 cagcaagggc ggcgagctat acatgcaagt cgagcggaca gatgggagct tgctccctga

61 tgttagcggc ggacgggtga gtaacacgtg ggtaacctgc ctgtaagact gggataactc

121 cgggaaaccg gggctaatac cggatggttg tttgaaccgc atggttcaaa cataaaaggt

181 ggcttcggct accacttaca gatggacccg cggcgcatta gctagttggt gaggtaacgg

241 ctcaccaagg caacgatgcg tagccgacct gagagggtga tcggccacac tgggactgag

301 acacggccca gactcctacg ggaggcagca gtagggaatc ttccgcaatg gacgaaagtc

361 tgacggagca acgccgcgtg agtgatgaag gttttcggat cgtaaagctc tgttgttagg

421 gaagaacaag taccgttcga atagggcggt accttgacgg tacctaacca gaaagccacg

481 gctaactacg tgccagcagc cgcggtaata cgtaggtggc aagcgttgtc cgggaattat

541 tgggcgtaaa gggctcgcag gcggtttctt aagtctgatg tgaaagcccc cggctcaacc

601 ggggagggtc attggaaact ggggaacttg agtgcagaag aggagagtgg aattccacgt

661 gtagcggtga aatgcgtaga gatgtggagg aacaccagtg gcgaaggcga ctctctggtc

721 tgtaactgac gctgaggagc gaaagcgtgg ggagcgaaca ggattagata ccctggtagt

781 ccacgccgta aacgatgagt gctaagtgtt agggggtttc cgccccttag tgctgcagct

841 aacgcattaa gcactccgcc tggggagtac ggtcgcaaga ctgaaactca aaggaattga

901 cgggggcccg cacaagcggt ggagcatgtg gtttaattcg aagcaacgcg aagaacctta

961 ccaggtcttg acatcctctg acaatcctag agataggacg tccccttcgg gggcagagtg

1021 acaggtggtg catggttgtc gtcagctcgt gtcgtgagat gttgggttaa gtcccgcaac

1081 gagcgcaacc cttgatctta gttgccagca ttcagttggg cactctaagg tgactgccgg

1141 tgacaaaccg gaggaaggtg gggatgacgt caaatcatca tgccccttat gacctgggct

1201 acacacgtgc tacaatggac agaacaaagg gcagcgaaac cgcgaggtta agccaatccc

1261 acaaatctgt tctcagttcg gatcgcagtc tgcaactcga ctgcgtgaag ctggaatcgc

1321 tagtaatcgc ggatcagcat gccgcggtga atacgttccc gggccttgta cacaccgccc

1381 gtcacaccac gagagtttgt aacacccgaa gtcggtgagg taacctttta ggagccagcc

1441 gccgaagctg acagattgtg

//

LOCUS PQ425627 1459 bp DNA linear BCT 04-OCT-2024

DEFINITION Bacillus sp. (in: firmicutes) strain C46 16S ribosomal RNA gene, partial sequence.

ACCESSION PQ425627

VERSION PQ425627

KEYWORDS .

SOURCE Bacillus sp. (in: firmicutes)

ORGANISM Bacillus sp. (in: firmicutes)

Bacteria; Bacillota; Bacilli; Bacillales; Bacillaceae; Bacillus.

REFERENCE 1 (bases 1 to 1459)

AUTHORS Huang,R.

TITLE Direct Submission

JOURNAL Submitted (04-OCT-2024) College of Animal Science and Technology,

Yunnan Agricultural University, Fengyuan Road No. 452, Kunming,

Yunnan 650201, China

COMMENT ##Assembly-Data-START##

Sequencing Technology :: Sanger dideoxy sequencing

##Assembly-Data-END##

FEATURES Location/Qualifiers

source 1..1459

/organism="Bacillus sp. (in: firmicutes)"

/mol_type="genomic DNA"

/strain="C46"

/db_xref="taxon:1409"

/geo_loc_name="China: Yunnan, Tengchong County"

/collection_date="May-2023"

rRNA <1..>1459

/product="16S ribosomal RNA"

ORIGIN

1 gaggaaatgg gcggcgtgct atacatgcaa gtcgagcgga cagatgggag cttgctccct

61 gatgttagcg gcggacgggt gagtaacacg tgggtaacct gcctgtaaga ctgggataac

121 tccgggaaac cggggctaat accggatggt tgtttgaacc gcatggttca aacataaaag

181 gtggcttcgg ctaccactta cagatggacc cgcggcgcat tagctagttg gtgaggtaac

241 ggctcaccaa ggcaacgatg cgtagccgac ctgagagggt gatcggccac actgggactg

301 agacacggcc cagactccta cgggaggcag cagtagggaa tcttccgcaa tggacgaaag

361 tctgacggag caacgccgcg tgagtgatga aggttttcgg atcgtaaagc tctgttgtta

421 gggaagaaca agtaccgttc gaatagggcg gtaccttgac ggtacctaac cagaaagcca

481 cggctaacta cgtgccagca gccgcggtaa tacgtaggtg gcaagcgttg tccggaatta

541 ttgggcgtaa agggctcgca ggcggtttct taagtctgat gtgaaagccc ccggctcaac

601 cggggagggt cattggaaac tggggaactt gagtgcagaa gaggagagtg gaattccacg

661 tgtagcggtg aaatgcgtag agatgtggag gaacaccagt ggcgaaggcg actctctggt

721 ctgtaactga cgctgaggag cgaaagcgtg gggagcgaac aggattagat accctggtag

781 tccacgccgt aaacgatgag tgctaagtgt tagggggttt ccgcccctta gtgctgcagc

841 taacgcatta agcactccgc ctggggagta cggtcgcaag actgaaactc aaaggaattg

901 acgggggccc gcacaagcgg tggagcatgt ggtttaattc gaagcaacgc gaagaacctt

961 accaggtctt gacatcctct gacaatccta gagataggac gtccccttcg ggggcagagt

1021 gacaggtggt gcatggttgt cgtcagctcg tgtcgtgaga tgttgggtta agtcccgcaa

1081 cgagcgcaac ccttgatctt agttgccagc attcagttgg gcactctaag gtgactgccg

1141 gtgacaaacc ggaggaaggt ggggatgacg tcaaatcatc atgcccctta tgacctgggc

1201 tacacacgtg ctacaatgga cagaacaaag ggcagcgaaa ccgcgaggtt aagccaatcc

1261 cacaaatctg ttctcagttc ggatcgcagt ctgcaactcg actgcgtgaa gctggaatcg

1321 ctagtaatcg cggatcagca tgccgcggtg aatacgttcc cgggccttgt acacaccgcc

1381 cgtcacacca cgagagtttg taacacccga agtcggtgag gtaacctttt aggagccagc

1441 cgccgaagtg acaagaagg

//

LOCUS PQ425628 1446 bp DNA linear BCT 04-OCT-2024

DEFINITION Erwinia billingiae strain C50 16S ribosomal RNA gene, partial sequence.

ACCESSION PQ425628

VERSION PQ425628

KEYWORDS .

SOURCE Erwinia billingiae

ORGANISM Erwinia billingiae

Bacteria; Pseudomonadota; Gammaproteobacteria; Enterobacterales;

Erwiniaceae; Erwinia.

REFERENCE 1 (bases 1 to 1446)

AUTHORS Huang,R.

TITLE Direct Submission

JOURNAL Submitted (04-OCT-2024) College of Animal Science and Technology,

Yunnan Agricultural University, Fengyuan Road No. 452, Kunming,

Yunnan 650201, China

COMMENT ##Assembly-Data-START##

Sequencing Technology :: Sanger dideoxy sequencing

##Assembly-Data-END##

FEATURES Location/Qualifiers

source 1..1446

/organism="Erwinia billingiae"

/mol_type="genomic DNA"

/strain="C50"

/db_xref="taxon:182337"

/geo_loc_name="China: Yunnan, Tengchong County"

/collection_date="May-2023"

rRNA <1..>1446

/product="16S ribosomal RNA"

ORIGIN

1 cgggatgggc ggcagctaca catgcagtcg aacggtagca cagagagctt gctcttgggt

61 gacgagtggc ggacgggtga gtaatgtctg ggaaactgcc tgatggaggg ggataactac

121 tggaaacggt agctaatacc gcataacgtc ttcggaccaa agtgggggac cttcgggcct

181 cacaccatcg gatgtgccca gatgggatta gctagtaggt ggggtaatgg ctcacctagg

241 cgacgatccc tagctggtct gagaggatga ccagccacac tggaactgag acacggtcca

301 gactcctacg ggaggcagca gtggggaata ttgcacaatg ggcgcaagcc tgatgcagcc

361 atgccgcgtg tatgaagaag gccttcgggt tgtaaagtac tttcagcggg gaggaaggcg

421 ataaggttaa taaccctgtc gattgacgtt acccgcagaa gaagcaccgg ctaactccgt

481 gccagcagcc gcggtaatac ggagggtgca agcgttaatc ggaattactg ggcgtaaagc

541 gcacgcaggc ggtctgtcaa gtcagatgtg aaatccccgg gcttaacctg ggaactgcat

601 ttgaaactgg caggctagag tcttgtagag gggggtagaa ttccaggtgt agcggtgaaa

661 tgcgtagaga tctggaggaa taccggtggc gaaggcggcc ccctggacaa agactgacgc

721 tcaggtgcga aagcgtgggg agcaaacagg attagatacc ctggtagtcc acgccgtaaa

781 cgatgtcgac ttggaggttg tgcccttgag gcgtggcttc cggagctaac gcgttaagtc

841 gaccgcctgg ggagtacggc cgcaaggtta aaactcaaat gaattgacgg gggcccgcac

901 aagcggtgga gcatgtggtt taattcgatg caacgcgaag aaccttacct gggccttgac

961 atccacggaa ttcggcagag atgccttagt gccttcggga accgtgagac aggtgctgca

1021 tggctgtcgt cagctcgtgt tgtgaaatgt tgggttaagt cccgcaacga gcgcaaccct

1081 tatcctttgt tgccagcgag taatgtcggg aactcaaagg agactgccgg tgacaaaccg

1141 gaggaaggtg gggatgacgt caagtcatca tggcccttac ggccagggct acacacgtgc

1201 tacaatggcg catacaaaga gaagcgaact cgcgagagca agcggacctc acaaagtgcg

1261 tcgtagtccg gatcggagtc tgcaactcga ctccgtgaag tcggaatcgc tagtaatcgt

1321 agatcagaat gctacggtga atacgttccc gggccttgta cacaccgccc gtcacaccat

1381 gggagtgggt tgcaaaagaa gtaggtagct taaccttcgg gagggcgcta ccactttgta

1441 tagtgg

//

LOCUS PQ425629 1466 bp DNA linear BCT 04-OCT-2024

DEFINITION Bacillus subtilis strain F1 16S ribosomal RNA gene, partial sequence.

ACCESSION PQ425629

VERSION PQ425629

KEYWORDS .

SOURCE Bacillus subtilis

ORGANISM Bacillus subtilis

Bacteria; Bacillota; Bacilli; Bacillales; Bacillaceae; Bacillus.

REFERENCE 1 (bases 1 to 1466)

AUTHORS Huang,R.

TITLE Direct Submission

JOURNAL Submitted (04-OCT-2024) College of Animal Science and Technology,

Yunnan Agricultural University, Fengyuan Road No. 452, Kunming,

Yunnan 650201, China

COMMENT ##Assembly-Data-START##

Sequencing Technology :: Sanger dideoxy sequencing

##Assembly-Data-END##

FEATURES Location/Qualifiers

source 1..1466

/organism="Bacillus subtilis"

/mol_type="genomic DNA"

/strain="F1"

/db_xref="taxon:1423"

/geo_loc_name="China: Yunnan, Tengchong County"

/collection_date="May-2023"

rRNA <1..>1466

/product="16S ribosomal RNA"

ORIGIN

1 agcaggcggg gtgctataca tgcaagtcga gcggacagat gggagcttgc tccctgatgt

61 tagcggcgga cgggtgagta acacgtgggt aacctgcctg taagactggg ataactccgg

121 gaaaccgggg ctaataccgg atggttgttt gaaccgcatg gttcaaacat aaaaggtggc

181 ttcggctacc acttacagat ggacccgcgg cgcattagct rgktgrtgag gtaamkcgct

241 caccmakgca wcgatgccgt agccgacctg agagggtgat ccgrccacac tggractgag

301 acacgrccca gactcctacg ggargcaggc agtagggaat cttccgcaat ggacgaaagt

361 ctgacggagc aacgccgcgt gagtgatgaa ggttttcgga tcgtaaagct ctgttgttag

421 ggaagaacaa gtaccgttcg aatagggcgg taccttgacg gtacctaacc agaaagccac

481 ggctaactac gtgccagcag ccgcggtaat acgtaggtgg caagcgttgt ccgggaatta

541 ttgggcgtaa agggctcgca ggcggtttct taagtctgat gtgaaagccc ccggctcaac

601 cggggagggt cattggaaac tggggaactt gagtgcagaa gaggagagtg gaattccacg

661 tgtagcggtg aaatgcgtag agatgtggag gaacaccagt ggcgaaggcg actctctggt

721 ctgtaactga cgctgaggag cgaaagcgtg gggagcgaac aggattagat accctggtag

781 tccacgccgt aaacgatgag tgctaagtgt tagggggttt ccgcccctta gtgctgcagc

841 taacgcatta agcactccgc ctggggagta cggtcgcaag actgaaactc aaaggaattg

901 acgggggccc gcacaagcgg tggagcatgt ggtttaattc gaagcaacgc gaagaacctt

961 accaggtctt gacatcctct gacaatccta gagataggac gtccccttcg ggggcagagt

1021 gacaggtggt gcatgggttg tcgtcagctc gtgtcgtgag atgttggggt taagtcccgc

1081 aacgagcgca accctttgat cttagttgcc agcattcagt tgggcacctc taaggtgact

1141 gccggtgaca aaccggagga aggtggggat gacgtcaaat catcatgccc cttatgacct

1201 gggctacaca ccggttgctt acaatggacm gaacaaaggg cagcgaaacc gcgaggttaa

1261 gccaatccca caaatctgtt ctcagttcgg atcgcagtct gcaactcgac tgcgtgaagc

1321 tggaatcgct agtaatcgcg gatcagcatg ccgcggtgaa tacgttcccg ggccttgtac

1381 acaccgcccg tcacaccacg agagtttgta acacccgaag tcggtgaggt aaccttttag

1441 gagccagccg ccgaaggtga cagagt

//

LOCUS PQ425630 1460 bp DNA linear BCT 04-OCT-2024

DEFINITION Bacillus subtilis strain F2 16S ribosomal RNA gene, partial sequence.

ACCESSION PQ425630

VERSION PQ425630

KEYWORDS .

SOURCE Bacillus subtilis

ORGANISM Bacillus subtilis

Bacteria; Bacillota; Bacilli; Bacillales; Bacillaceae; Bacillus.

REFERENCE 1 (bases 1 to 1460)

AUTHORS Huang,R.

TITLE Direct Submission

JOURNAL Submitted (04-OCT-2024) College of Animal Science and Technology,

Yunnan Agricultural University, Fengyuan Road No. 452, Kunming,

Yunnan 650201, China

COMMENT ##Assembly-Data-START##

Sequencing Technology :: Sanger dideoxy sequencing

##Assembly-Data-END##

FEATURES Location/Qualifiers

source 1..1460

/organism="Bacillus subtilis"

/mol_type="genomic DNA"

/strain="F2"

/db_xref="taxon:1423"

/geo_loc_name="China: Yunnan, Tengchong County"

/collection_date="May-2023"

rRNA <1..>1460

/product="16S ribosomal RNA"

ORIGIN

1 cgcgtggcgg gtgctataca tgcaagtcga gcggacagat gggagcttgc tccctgatgt

61 tagcggcgga cgggtgagta acacgtgggt aacctgcctg taagactggg ataactccgg

121 gaaaccgggg ctaataccgg atggttgttt gaaccgcatg gttcaaacat aaaaggtggc

181 ttcggctacc acttacagat ggacccgcgg cgcattagct agttggtgag gtaacggctc

241 acmarggcaa tcgatgcgta gcsgacctga ggagggtgat cgrccacacy kkgactgaga

301 cacgrcccag actcctacsg gaggcagcag tagggaatcy tccgcmatgg acgraagtct

361 gacggagcaa cgccgcgtga gtgatgaagg ttttcggatc gtaaagctct gttgttaggg

421 aagaacaagt accgttcgaa tagggcggta ccttgacggt acctaaccag aaagccacgg

481 ctaactacgt gccagcagcc gcggtaatac gtaggtggca agcgttgtcc ggaattattg

541 ggcgtaaagg gctcgcaggc ggtttcttaa gtctgatgtg aaagcccccg gctcaaccgg

601 ggagggtcat tggaaactgg ggaacttgag tgcagaagag gagagtggaa ttccacgtgt

661 agcggtgaaa tgcgtagaga tgtggaggaa caccagtggc gaaggcgact ctctggtctg

721 taactgacgc tgaggagcga aagcgtgggg agcgaacagg attagatacc ctggtagtcc

781 acgccgtaaa cgatgagtgc taagtgttag ggggtttccg ccccttagtg ctgcagctaa

841 cgcattaagc actccgcctg gggagtacgg tcgcaagact gaaactcaaa ggaattgacg

901 ggggcccgca caagcggtgg agcatgtggt ttaattcgaa gcaacgcgaa gaaccttacc

961 aggtcttgac atcctctgac aatcctagag ataggacgtc cccttcgggg gcagagtgac

1021 aggtggtgca tggttgtcgt cagctcgtgt cgtgagatgt tgggttaagt cccgcaacga

1081 gcgcaaccct tgatcttagt tgccagcatt cagttgggca cctctaaggt gactgccggt

1141 gacaaaccgg aggaaggtgg ggatgacgtc maatcatcat gccccttatg amctgggcta

1201 caccacgtgc tacawtggac caggaacaca arkgcagcsa amscgcgagg ttaagccaat

1261 cccacaaatc tgttctcagt tcggatcgca gtctgcaact cgactgcgtg aagctggaat

1321 cgctagtaat cgcggatcag catgccgcgg tgaatacgtt cccgggcctt gtacacaccg

1381 cccgtcacac cacgagagtt tgtaacaccc gaagtcggtg aggtaacctt ttaggagcca

1441 gccgccgaag gtgacagagg

//

LOCUS PQ425631 1461 bp DNA linear BCT 04-OCT-2024

DEFINITION Bacillus licheniformis strain F4 16S ribosomal RNA gene, partial sequence.

ACCESSION PQ425631

VERSION PQ425631

KEYWORDS .

SOURCE Bacillus licheniformis

ORGANISM Bacillus licheniformis

Bacteria; Bacillota; Bacilli; Bacillales; Bacillaceae; Bacillus.

REFERENCE 1 (bases 1 to 1461)

AUTHORS Huang,R.

TITLE Direct Submission

JOURNAL Submitted (04-OCT-2024) College of Animal Science and Technology,

Yunnan Agricultural University, Fengyuan Road No. 452, Kunming,

Yunnan 650201, China

COMMENT ##Assembly-Data-START##

Sequencing Technology :: Sanger dideoxy sequencing

##Assembly-Data-END##

FEATURES Location/Qualifiers

source 1..1461

/organism="Bacillus licheniformis"

/mol_type="genomic DNA"

/strain="F4"

/db_xref="taxon:1402"

/geo_loc_name="China: Yunnan, Tengchong County"

/collection_date="May-2023"

rRNA <1..>1461

/product="16S ribosomal RNA"

ORIGIN

1 cggcatggcg gcagctatac atgcaagtcg agcggaccga cgggagcttg ctcccttagg

61 tcagcggcgg acgggtgagt aacacgtggg taacctgcct gtaagactgg gataactccg

121 ggaaaccggg gctaataccg gatgcttgat tgaaccgcat ggttcaatta taaaaggtgg

181 cttttagcta ccacttacag atggacccgc ggcgcattag ctagttggtg aggtaacggc

241 tcaccaaggc gacgatgcgt agccgacctg agagggtgat cggccacact gggactgagg

301 acacgkccca gactcctacg ggargcagca gtagggaatc ttccgcaatg gacgaaagtc

361 tgacggagca acgccgcgtg agtgatgaag gttttcggat cgtaaaactc tgttgttagg

421 gaagaacaag taccgttcga atagggcggt accttgacgg tacctaacca gaaagccacg

481 gctaactacg tgccagcagc cgcggtaata cgtaggtggc aagcgttgtc cgggaattat

541 tgggcgtaaa gcgcgcgcag gcggtttctt aagtctgatg tgaaagcccc cggctcaacc

601 ggggagggtc attggaaact ggggaacttg agtgcagaag aggagagtgg aattccacgt

661 gtagcggtga aatgcgtaga gatgtggagg aacaccagtg gcgaaggcga ctctctggtc

721 tgtaactgac gctgaggcgc gaaagcgtgg ggagcgaaca ggattagata ccctggtagt

781 ccacgccgta aacgatgagt gctaagtgtt agagggtttc cgccctttag tgctgcagca

841 aacgcattaa gcactccgcc tggggagtac ggtcgcaaga ctgaaactca aaggaattga

901 cgggggcccg cacaagcggt ggagcatgtg gtttaattcg aagcaacgcg aagaacctta

961 ccaggtcttg acatcctctg acaaccctag agatagggct tccccttcgg gggcagagtg

1021 acaggtggtg catggttgtc gtcagctcgt gtcgtgagat gttgggktaa gtcccgcaac

1081 gagcgcaacc cttgatctta gttgccagca ttcagttggg cacctctaag gtgactgccg

1141 gtgacaaacc ggaggarggt gggggatgac gtcmaatcat catgsccctt aatgamctgg

1201 gcctacacca cggtgcctac aatgsgcaga acaaagggca gcgaagccgc gaggctaagc

1261 caatcccaca aatctgttct cagttcggat cgcagtctgc aactcgactg cgtgaagctg

1321 gaatcgctag taatcgcgga tcagcatgcc gcggtgaata cgttcccggg ccttgtacac

1381 accgcccgtc acaccacgag agtttgtaac acccgaagtc ggtgaggtaa cctttggagc

1441 cagccgccga agtgacagag g

//

LOCUS PQ425632 1443 bp DNA linear BCT 04-OCT-2024

DEFINITION Pseudomonas tolaasii strain F6 16S ribosomal RNA gene, partial sequence.

ACCESSION PQ425632

VERSION PQ425632

KEYWORDS .

SOURCE Pseudomonas tolaasii

ORGANISM Pseudomonas tolaasii

Bacteria; Pseudomonadota; Gammaproteobacteria; Pseudomonadales;

Pseudomonadaceae; Pseudomonas.

REFERENCE 1 (bases 1 to 1443)

AUTHORS Huang,R.

TITLE Direct Submission

JOURNAL Submitted (04-OCT-2024) College of Animal Science and Technology,

Yunnan Agricultural University, Fengyuan Road No. 452, Kunming,

Yunnan 650201, China

COMMENT ##Assembly-Data-START##

Sequencing Technology :: Sanger dideoxy sequencing

##Assembly-Data-END##

FEATURES Location/Qualifiers

source 1..1443

/organism="Pseudomonas tolaasii"

/mol_type="genomic DNA"

/strain="F6"

/db_xref="taxon:29442"

/geo_loc_name="China: Yunnan, Tengchong County"

/collection_date="May-2023"

rRNA <1..>1443

/product="16S ribosomal RNA"

ORIGIN

1 ggcatggcgg cagctacaca tgcaagtcga gcggtagaga gaagcttgct tctcttgaga

61 gcggcggacg ggtgagtaat gcctaggaat ctgcctggta gtgggggata acgttcggaa

121 acggacgcta ataccgcata cgtcctacgg gagaaagcag gggaccttcg ggccttgcgc

181 tatcagatga gcctaggtcg gattagctag ttggtgaggt aatggctcac caaggcgacg

241 atccgtaact ggtctgagag gatgatcagt cacactggaa ctgagaacac sgtccagact

301 cctacsggag gcagcagtgg ggaatattgg acaatgggcg aaagcctgat tccagccatg

361 ccgcgtgtgt gaagraggtc ttcggattgt aaagcacctt taagttggga ggaagggcag

421 ttgcctaata cgtarctgtt ttgacgttac cgacagaata agcaccggct aactctgtgc

481 cagcagccgc ggtaatacag agggtgcaag cgttaatcgg aattactggg cgtaaagcgc

541 gcgtaggtgg tttgttaagt tggatgtgaa atccccgggc tcaacctggg aactgcattc

601 aaaactgact gactagagta tggtagaggg tggtggaatt tcctgtgtag cggtgaaatg

661 cgtagatata ggaaggaaca ccagtggcga aggcgaccac ctggactgat actgacactg

721 aggtgcgaaa gcgtggggag caaacaggat tagataccct ggtagtccac gccgtaaacg

781 atgtcaacta gccgttggaa gccttgagct tttagtggcg cagctaacgc attaagttga

841 ccgcctgggg agtacggccg caaggttaaa actcaaatga attgacgggg gcccgcacaa

901 gcggtggagc atgtggttta attcgaagca acgcgaagaa ccttaccagg ccttgacatc

961 caatgaactt tcyagagatg gattggtgcc ttcgggaaca ttgagacagg tgctgcatgg

1021 ctgtcgtcag ctcgtgtcgt gagatgttgg gttaagtccc gtaacgagcg caacccttgt

1081 ccttagttac cagcacgtaa tggtkggcac ctctaaggag actgccggtg acaaaccgga

1141 ggaaggtggg gatgacgtca agtcatcatg gsccttacgg cctgggctac acacgtgcta

1201 caatggtcgg tacaaagggt tgccaagccg cgaggtggag ctaatcccat aaaaccgatc

1261 gtagtccgga tcgcagtctg caactcgact gcgtgaagtc ggaatcgcta gtaatcgcga

1321 atcagaatgt cgcggtgaat acgttcccgg gccttgtaca caccgcccgt cacaccatgg

1381 gagtgggttg caccagaagt agctagtcta accttcggga ggacggtacc acggtgatcg

1441 ggc

//

LOCUS PQ425633 1458 bp DNA linear BCT 04-OCT-2024

DEFINITION Bacillus subtilis strain F9 16S ribosomal RNA gene, partial sequence.

ACCESSION PQ425633

VERSION PQ425633

KEYWORDS .

SOURCE Bacillus subtilis

ORGANISM Bacillus subtilis

Bacteria; Bacillota; Bacilli; Bacillales; Bacillaceae; Bacillus.

REFERENCE 1 (bases 1 to 1458)

AUTHORS Huang,R.

TITLE Direct Submission

JOURNAL Submitted (04-OCT-2024) College of Animal Science and Technology,

Yunnan Agricultural University, Fengyuan Road No. 452, Kunming,

Yunnan 650201, China

COMMENT ##Assembly-Data-START##

Sequencing Technology :: Sanger dideoxy sequencing

##Assembly-Data-END##

FEATURES Location/Qualifiers

source 1..1458

/organism="Bacillus subtilis"

/mol_type="genomic DNA"

/strain="F9"

/db_xref="taxon:1423"

/geo_loc_name="China: Yunnan, Tengchong County"

/collection_date="May-2023"

rRNA <1..>1458

/product="16S ribosomal RNA"

ORIGIN

1 gggaagtgcg gtgctataca tgcagtcgag cggacagatg ggagcttgct ccctgatgtt

61 agcggcggac gggtgagtaa cacgtgggta acctgcctgt aagactggga taactccggg

121 aaaccggggc taataccgga tggttgtttg aaccgcatgg ttcaaacata aaaggtggct

181 tcggctacca cttacagatg gaccckmggc gcattarcta gttggtgagc gtaaysgctc

241 acctawggca atcgattgcg tagccgacct garrgggtga tcsrccacac tggractgag

301 acacgkccca gactcctacg ggagscaggc agtagggaat cttccgcaat ggacgaaagt

361 ctgacggagc aacgccgcgt gagtgatgaa ggttttcgga tcgtaaagct ctgttgttag

421 ggaagaacaa gtaccgttcg aatagggcgg taccttgacg gtacctaacc agaaagccac

481 ggctaactac gtgccagcag ccgcggtaat acgtaggtgg caagcgttgt ccggaattat

541 tgggcgtaaa gggctcgcag gcggtttctt aagtctgatg tgaaagcccc cggctcaacc

601 ggggagggtc attggaaact ggggaacttg agtgcagaag aggagagtgg aattccacgt

661 gtagcggtga aatgcgtaga gatgtggagg aacaccagtg gcgaaggcga ctctctggtc

721 tgtaactgac gctgaggagc gaaagcgtgg ggagcgaaca ggattagata ccctggtagt

781 ccacgccgta aacgatgagt gctaagtgtt agggggtttc cgccccttag tgctgcagct

841 aacgcattaa gcactccgcc tggggagtac ggtcgcaaga ctgaaactca aaggaattga

901 cgggggcccg cacaagcggt ggagcatgtg gtttaattcg aagcaacgcg aagaacctta

961 ccaggtcttg acatcctctg acaatcctag agataggacg tccccttcgg gggcagagtg

1021 acaggtggtg catggttgtc gtcagctcgt gtcgtgagat gttgggktaa gtcccgcaac

1081 gagcgcaacc cttgatctta gttgccagca ttcagttggg cacctctaag gtgactgccg

1141 gtgacaaacc ggargaaggt ggggatgacg tcaaatcatc atgcccctta tgacctgggc

1201 ctacacacgt gctacaatgg acagaacaaa gggcagcgaa accgcgaggt taagccaatc

1261 ccacaaatct gttctcagtt cggatcgcag tctgcaactc gactgcgtga agctggaatc

1321 gctagtaatc gcggatcagc atgccgcggt gaatacgttc ccgggccttg tacacaccgc

1381 ccgtcacacc acgagagttt gtaacacccg aagtcggtga ggtaaccttt taggagccag

1441 ccgccgaagg tgacagag

//

LOCUS PQ425634 1461 bp DNA linear BCT 04-OCT-2024

DEFINITION Bacillus licheniformis strain F12 16S ribosomal RNA gene, partial sequence.

ACCESSION PQ425634

VERSION PQ425634

KEYWORDS .

SOURCE Bacillus licheniformis

ORGANISM Bacillus licheniformis

Bacteria; Bacillota; Bacilli; Bacillales; Bacillaceae; Bacillus.

REFERENCE 1 (bases 1 to 1461)

AUTHORS Huang,R.

TITLE Direct Submission

JOURNAL Submitted (04-OCT-2024) College of Animal Science and Technology,

Yunnan Agricultural University, Fengyuan Road No. 452, Kunming,

Yunnan 650201, China

COMMENT ##Assembly-Data-START##

Sequencing Technology :: Sanger dideoxy sequencing

##Assembly-Data-END##

FEATURES Location/Qualifiers

source 1..1461

/organism="Bacillus licheniformis"

/mol_type="genomic DNA"

/strain="F12"

/db_xref="taxon:1402"

/geo_loc_name="China: Yunnan, Tengchong County"

/collection_date="May-2023"

rRNA <1..>1461

/product="16S ribosomal RNA"

ORIGIN

1 gcatggcggg tgctatacat gcaagtcgag cggaccgacg ggagcttgct cccttaggtc

61 agcggcggac gggtgagtaa cacgtgggta acctgcctgt aagactggga taactccggg

121 aaaccggggc taataccgga tgcttgattg aaccgcatgg ttcaattata aaaggtggct

181 tttagctacc acttacagat ggacccgcgg cgcakymkct aagttgggtc gaggtawcgg

241 ctcaccwagg cgacgatgcg tagccgacct grragggtra tcsgccacac tgggactgag

301 acacgrccca gactcctacg ggaggccagc agtagggaat cttccgcaat ggacgaaagt

361 ctgacggagc aacgccgcgt gagtgatgaa ggttttcgga tcgtaaaact ctgttgttag

421 ggaagaacaa gtaccgttcg aatagggcgg taccttgacg gtacctaacc agaaagccac

481 ggctaactac gtgccagcag ccgcggtaat acgtaggtgg caagcgttgt ccgggaatta

541 ttgggcgtaa agcgcgcgca ggcggtttct taagtctgat gtgaaagccc ccggctcaac

601 cggggagggt cattggaaac tggggaactt gagtgcagaa gaggagagtg gaattccacg

661 tgtagcggtg aaatgcgtag agatgtggag gaacaccagt ggcgaaggcg actctctggt

721 ctgtaactga cgctgaggcg cgaaagcgtg gggagcgaac aggattagat accctggtag

781 tccacgccgt aaacgatgag tgctaagtgt tagagggttt ccgcccttta gtgctgcagc

841 aaacgcatta agcactccgc ctggggagta cggtcgcaag actgaaactc aaaggaattg

901 acgggggccc gcacaagcgg tggagcatgt ggtttaattc gaagcaacgc gaagaacctt

961 accaggtctt gacatcctct gacaacccta gagatagggc ttccccttcg ggggcagagt

1021 gacaggtggt gcatggttgt cgtcagctcg tgtcgtgaga tgttgggtta agtcccgcaa

1081 cgagcgcaac ccttgatctt agttgscagc attcagttgg gcacctctaa ggtgactgcc

1141 ggtgacaaac cggaggaagg tggggatgac gtcmaatcat catgcccctt atgacctggg

1201 ctacacacgt gctacaatgg gccagaacma agggcagcga aagctcgcga gaggctwagm

1261 saatcccaca aatctgttct cagttcggat cgcagtctgc aactcgactg cgtgaagctg

1321 gaatcgctag taatcgcgga tcagcatgcc gcggtgaata cgttcccggg ccttgtacac

1381 accgcccgtc acaccacgag agtttgtaac acccgaagtc ggtgaggtaa ccttttggag

1441 ccagccgccg aaggtgacag a

//

LOCUS PQ425635 1459 bp DNA linear BCT 04-OCT-2024

DEFINITION Bacillus sp. (in: firmicutes) strain F13 16S ribosomal RNA gene, partial sequence.

ACCESSION PQ425635

VERSION PQ425635

KEYWORDS .

SOURCE Bacillus sp. (in: firmicutes)

ORGANISM Bacillus sp. (in: firmicutes)

Bacteria; Bacillota; Bacilli; Bacillales; Bacillaceae; Bacillus.

REFERENCE 1 (bases 1 to 1459)

AUTHORS Huang,R.

TITLE Direct Submission

JOURNAL Submitted (04-OCT-2024) College of Animal Science and Technology,

Yunnan Agricultural University, Fengyuan Road No. 452, Kunming,

Yunnan 650201, China

COMMENT ##Assembly-Data-START##

Sequencing Technology :: Sanger dideoxy sequencing

##Assembly-Data-END##

FEATURES Location/Qualifiers

source 1..1459

/organism="Bacillus sp. (in: firmicutes)"

/mol_type="genomic DNA"

/strain="F13"

/db_xref="taxon:1409"

/geo_loc_name="China: Yunnan, Tengchong County"

/collection_date="May-2023"

rRNA <1..>1459

/product="16S ribosomal RNA"

ORIGIN

1 gcatgcgggg tgctatacat gcagtcgagc ggacagatgg gagcttgctc cctgatgtta

61 gcggcggacg ggtgagtaac acgtgggtaa cctgcctgta agactgggat aactccggga

121 aaccggggct aataccggat ggttgtttga accgcatggt tcaaacataa aaggtggctt

181 cggctaccac ttacagatgg acccgcggcg catwrgctag gttgkygagg gtarcgrctc

241 accaasgcaa cgatgcgtag ccgacctgag arggtgatcg gccacactgg gactgagaca

301 cgkcccagac tcctacggga ggccagcagt agggaatctt ccgcaatgga cgaaagtctg

361 acggagcaac gccgcgtgag tgatgaaggt tttcggatcg taaagctctg ttgttaggga

421 agaacaagta ccgttcgaat agggcggtac cttgacggta cctaaccaga aagccacggc

481 taactacgtg ccagcagccg cggtaatacg taggtggcaa gcgttgtccg gaattattgg

541 gcgtaaaggg ctcgcaggcg gtttcttaag tctgatgtga aagcccccgg ctcaaccggg

601 gagggtcatt ggaaactggg gaacttgagt gcagaagagg agagtggaat tccacgtgta

661 gcggtgaaat gcgtagagat gtggaggaac accagtggcg aaggcgactc tctggtctgt

721 aactgacgct gaggagcgaa agcgtgggga gcgaacagga ttagataccc tggtagtcca

781 cgccgtaaac gatgagtgct aagtgttagg gggtttccgc cccttagtgc tgcagctaac

841 gcattaagca ctccgcctgg ggagtacggt cgcaagactg aaactcaaag gaattgacgg

901 gggcccgcac aagcggtgga gcatgtggtt taattcgaag caacgcgaag aaccttacca

961 ggtcttgaca tcctctgaca atcctagaga taggacgtcc ccttcggggg cagagtgaca

1021 ggtggtgcat gggttgtcgt cagctcgtgt cgtgagatgt tgggttaagt cccgcaacga

1081 gcgcaaccyt tgatcttagt tgcyagcatt cagttgggca ctctaaaggt gactgccggt

1141 gacaaaccgg aggaaggtgg ggatgacgtc aaatcatcat gycccttatg gacctkggct

1201 tacacacgtg ctacaattgr acrgaacmaa gggcaagcgt aaaccgcsag gttaagccaa

1261 tcccacaaat ctgttctcag ttcggatcgc agtctgcaac tcgactgcgt gaagctggaa

1321 tcgctagtaa tcgcggatca gcatgccgcg gtgaatacgt tcccgggcct tgtacacacc

1381 gcccgtcaca ccacgagagt ttgtaacacc cgaagtcggt gaggtaacct ttaggagcca

1441 gccgccgaag gtggaccgg

//

LOCUS PQ425636 1475 bp DNA linear BCT 04-OCT-2024

DEFINITION Bacillus sp. (in: firmicutes) strain F14 16S ribosomal RNA gene, partial sequence.

ACCESSION PQ425636

VERSION PQ425636

KEYWORDS .

SOURCE Bacillus sp. (in: firmicutes)

ORGANISM Bacillus sp. (in: firmicutes)

Bacteria; Bacillota; Bacilli; Bacillales; Bacillaceae; Bacillus.

REFERENCE 1 (bases 1 to 1475)

AUTHORS Huang,R.

TITLE Direct Submission

JOURNAL Submitted (04-OCT-2024) College of Animal Science and Technology,

Yunnan Agricultural University, Fengyuan Road No. 452, Kunming,

Yunnan 650201, China

COMMENT ##Assembly-Data-START##

Sequencing Technology :: Sanger dideoxy sequencing

##Assembly-Data-END##

FEATURES Location/Qualifiers

source 1..1475

/organism="Bacillus sp. (in: firmicutes)"

/mol_type="genomic DNA"

/strain="F14"

/db_xref="taxon:1409"

/geo_loc_name="China: Yunnan, Tengchong County"

/collection_date="May-2023"

rRNA <1..>1475

/product="16S ribosomal RNA"

ORIGIN

1 ggcatggcgg ggtgctatac atgcaagtcg agcggacaga tgggagcttg ctccctgatg

61 ttagcggcgg acgggtgagt aacacgtggg taacctgcct gtaagactgg gataactccg

121 ggaaaccggg gctaataccg gatggttgtt tgaaccgcat ggttcaaaca taaaaggtgg

181 cttcggctac cacttacaga tggacccgcg gcgcattagc tagttggtga ggtaacggct

241 caccaaggca acgatgccgt aggcccgacc ttgrgagggt gatcggccac cactgggact

301 gagacacgrc ccagactcct acsggargca gcagtaggga atcttccgca atggacgaaa

361 gtctgacgga gcaacgccgc gtgaggtgat gaaggttttc ggatcgtaaa gctctgttgt

421 tagggaagaa caagtaccgt tcgaataggg cggtaccttg acggtaccta accagaaagc

481 cacggctaac tacgtgccag cagccgcggt aatacgtagg tggcaagcgt tgtccgggaa

541 ttattgggcg taaagggctc gcaggcggtt tcttaagtct gatgtgaaag cccccggctc

601 aaccggggag ggtcattgga aactggggaa cttgagtgca gaagaggaga gtggaattcc

661 acgtgtagcg gtgaaatgcg tagagatgtg gaggaacacc agtggcgaag gcgactctct

721 ggtctgtaac tgacgctgag gagcgaaagc gtggggagcg aacaggatta gataccctgg

781 tagtccacgc cgtaaacgat gagtgctaag tgttaggggg tttccgcccc ttagtgctgc

841 agctaacgca ttaagcactc cgcctgggga gtacggtcgc aagactgaaa ctcaaaggaa

901 ttgacggggg cccgcacaag cggtggagca tgtggtttaa ttcgaagcaa cgcgaagaac

961 cttaccaggt cttgacatcc tctgacaatc ctagagatag gacgtcccct tcgggggcag

1021 aagtgacagg tggtgcatgg gttgtcgtca gctcgtgtcg tgagatgttg ggttaagtcc

1081 cgcaacgagc gcaacccttg atcttagttg ccagcattca gttggsmact ctaaaggtga

1141 ctgccggtga acaaaccgga grarggtggg gatgacgtca aatcatcatg ccccttaatg

1201 aacctggggc ctacacaaac ggtgcctaca caatggacag aacaaagggc agcgaaaccg

1261 cgaggttaag ccaatcccac aaatctgttc tcagttcgga tcgcagtctg caactcgact

1321 gcgtgaagct ggaatcgcta gtaatcgcgg atcagcatgc cgcggtgaat acgttcccgg

1381 gccttgtaca caccgcccgt cacaccacga gagtttgtaa cacccgaagt cggtgaggta

1441 accttttagg agccagccgc cgaagtgaac agatg

//

LOCUS PQ425637 1463 bp DNA linear BCT 04-OCT-2024

DEFINITION Bacillus subtilis strain F15 16S ribosomal RNA gene, partial sequence.

ACCESSION PQ425637

VERSION PQ425637

KEYWORDS .

SOURCE Bacillus subtilis

ORGANISM Bacillus subtilis

Bacteria; Bacillota; Bacilli; Bacillales; Bacillaceae; Bacillus.

REFERENCE 1 (bases 1 to 1463)

AUTHORS Huang,R.

TITLE Direct Submission

JOURNAL Submitted (04-OCT-2024) College of Animal Science and Technology,

Yunnan Agricultural University, Fengyuan Road No. 452, Kunming,

Yunnan 650201, China

COMMENT ##Assembly-Data-START##

Sequencing Technology :: Sanger dideoxy sequencing

##Assembly-Data-END##

FEATURES Location/Qualifiers

source 1..1463

/organism="Bacillus subtilis"

/mol_type="genomic DNA"

/strain="F15"

/db_xref="taxon:1423"

/geo_loc_name="China: Yunnan, Tengchong County"

/collection_date="May-2023"

rRNA <1..>1463

/product="16S ribosomal RNA"

ORIGIN

1 gggatgcggg tgctatacat gcaagtcgag cggacagatg ggagcttgct ccctgatgtt

61 agcggcggac gggtgagtaa cacgtgggta acctgcctgt aagactggga taactccggg

121 aaaccggggc taataccgga tggttgtttg aaccgcatgg ttcaaacata aaaggtggct

181 tcggctacca cttacagatg gacccgcggc gcattagtct agttgggtgg aagktaacgg

241 tctcaccmag gcaacgaatg cgtagccgac ctgagaggkk gatcsgmcac actggractg

301 agacacgkcc cagactccta cgggaggcag cagtagggaa tcttccgcaa tggacgaaag

361 tctgacggag caacgccgcg tgagtgatga aggttttcgg atcgtaaagc tctgttgtta

421 gggaagaaca agtaccgttc gaatagggcg gtaccttgac ggtacctaac cagaaagcca

481 cggctaacta cgtgccagca gccgcggtaa tacgtaggtg gcaagcgttg tccggaatta

541 ttgggcgtaa agggctcgca ggcggtttct taagtctgat gtgaaagccc ccggctcaac

601 cggggagggt cattggaaac tggggaactt gagtgcagaa gaggagagtg gaattccacg

661 tgtagcggtg aaatgcgtag agatgtggag gaacaccagt ggcgaaggcg actctctggt

721 ctgtaactga cgctgaggag cgaaagcgtg gggagcgaac aggattagat accctggtag

781 tccacgccgt aaacgatgag tgctaagtgt tagggggttt ccgcccctta gtgctgcagc

841 taacgcatta agcactccgc ctggggagta cggtcgcaag actgaaactc aaaggaattg

901 acgggggccc gcacaagcgg tggagcatgt ggtttaattc gaagcaacgc gaagaacctt

961 accaggtctt gacatcctct gacaatccta gagataggac gtccccttcg ggggcagagt

1021 gacaggtggt gcatgggttg tcgtcagctc gtgtcgtgag atgttgggkt wagtcccgca

1081 acgagcgcaa cccttgatct tagttgccag cattcagttg ggcacctcta aggtgactgc

1141 cggtgacaaa ccggaggaag gtgggratga cgtcaaatca tcatgcccyt tatgaccttg

1201 ggctwcacac gtgcttacaa tkgacagaaa caaagggcag csraaccscs aggttaagcc

1261 aatcccacaa atctgttctc agttcggatc gcagtctgca actcgactgc gtgaagctgg

1321 aatcgctagt aatcgcggat cagcatgccg cggtgaatac gttcccgggc cttgtacaca

1381 ccgcccgtca caccacgaga gtttgtaaca cccgaagtcg gtgaggtaac cttttaggag

1441 ccagccgccg aagtgaccag agg

//

LOCUS PQ425638 1458 bp DNA linear BCT 04-OCT-2024

DEFINITION Bacillus subtilis strain F22 16S ribosomal RNA gene, partial sequence.

ACCESSION PQ425638

VERSION PQ425638

KEYWORDS .

SOURCE Bacillus subtilis

ORGANISM Bacillus subtilis

Bacteria; Bacillota; Bacilli; Bacillales; Bacillaceae; Bacillus.

REFERENCE 1 (bases 1 to 1458)

AUTHORS Huang,R.

TITLE Direct Submission

JOURNAL Submitted (04-OCT-2024) College of Animal Science and Technology,

Yunnan Agricultural University, Fengyuan Road No. 452, Kunming,

Yunnan 650201, China

COMMENT ##Assembly-Data-START##

Sequencing Technology :: Sanger dideoxy sequencing

##Assembly-Data-END##

FEATURES Location/Qualifiers

source 1..1458

/organism="Bacillus subtilis"

/mol_type="genomic DNA"

/strain="F22"

/db_xref="taxon:1423"

/geo_loc_name="China: Yunnan, Tengchong County"

/collection_date="May-2023"

rRNA <1..>1458

/product="16S ribosomal RNA"

ORIGIN

1 agccctgcgg gtgctataca tgcagtcgag cggacagatg ggagcttgct ccctgatgtt

61 agcggcggac gggtgagtaa cacgtgggta acctgcctgt aagactggga taactccggg

121 aaaccggggc taataccgga tggttgtttg aaccgcatgg ttcaaacata aaaggtggct

181 tcggctacca cttacagatg gacccgcggc gcattagcta gytggtkasg taaysgytca

241 cacmaggcaw cgatgcgtag ccgacctgag agggtgatcg gccacactgg ractgagaca

301 cggyccagac tcctacsgga ggcagcagta rggratcttc cgcaatggac gaaagtctga

361 cggagcamcg ccgcgtgagt gatgaaggtt ttcggatcgt aaagctctgt tgttagggaa

421 gaacaagtac cgttcgaata gggcggtacc ttgacggtac ctaacccaga aagccacggc

481 taactacgtg ccagcagccg cggtaatacg taggtggcaa gcgttgtccg gaattattgg

541 gcgtaaaggg ctcgcaggcg gtttcttaag tctgatgtga aagcccccgg ctcaaccggg

601 gagggtcatt ggaaactggg gaacttgagt gcagaagagg agagtggaat tccacgtgta

661 gcggtgaaat gcgtagagat gtggaggaac accagtggcg aaggcgactc tctggtctgt

721 aactgacgct gaggagcgaa agcgtgggga gcgaacagga ttagataccc tggtagtcca

781 cgccgtaaac gatgagtgct aagtgttagg gggtttccgc cccttagtgc tgcagctaac

841 gcattaagca ctccgcctgg ggagtacggt cgcaagactg aaactcaaag gaattgacgg

901 gggcccgcac aagcggtgga gcatgtggtt taattcgaag caacgcgaag aacctttacc

961 aggtcttgac atcctctgac aatcctagag ataggacgtc cctcttcggg ggcagagtga

1021 caggtggtgc atgggttgtc gtcagctcgt gtcgtgagat gttgggktwa gtcccgcaac

1081 gagcgcaacc cttgatctta gttgccagca ttcagttggg cactctaagg tgactgccgg

1141 tgacaaaccg gaggaaggtg gggatgacgt cmaatcatca tgccccttat gamctgggct

1201 acacacgtgc tacaatggac rgaacaamgg gccagcgaam cgcgcgaggt twagccaatc

1261 ccacaaatct gttctcagtt cggatcgcag tctgcaactc gactgcgtga agctggaatc

1321 gctagtaatc gcggatcagc atgccgcggt gaatacgttc ccgggccttg tacacaccgc

1381 ccgtcacacc acgagagttt gtaacacccg aagtcggtga ggtaaccttt taggagccag

1441 ccgccgaagt gacagagg

//

LOCUS PQ425639 1460 bp DNA linear BCT 04-OCT-2024

DEFINITION Bacillus subtilis strain F36 16S ribosomal RNA gene, partial sequence.

ACCESSION PQ425639

VERSION PQ425639

KEYWORDS .

SOURCE Bacillus subtilis

ORGANISM Bacillus subtilis

Bacteria; Bacillota; Bacilli; Bacillales; Bacillaceae; Bacillus.

REFERENCE 1 (bases 1 to 1460)

AUTHORS Huang,R.

TITLE Direct Submission

JOURNAL Submitted (04-OCT-2024) College of Animal Science and Technology,

Yunnan Agricultural University, Fengyuan Road No. 452, Kunming,

Yunnan 650201, China

COMMENT ##Assembly-Data-START##

Sequencing Technology :: Sanger dideoxy sequencing

##Assembly-Data-END##

FEATURES Location/Qualifiers

source 1..1460

/organism="Bacillus subtilis"

/mol_type="genomic DNA"

/strain="F36"

/db_xref="taxon:1423"

/geo_loc_name="China: Yunnan, Tengchong County"

/collection_date="May-2023"

rRNA <1..>1460

/product="16S ribosomal RNA"

ORIGIN

1 accagtgcgg cgtgctatac atgcagtcga gcggacagat gggagcttgc tccctgatgt

61 tagcggcgga cgggtgagta acacgtgggt aacctgcctg taagactggg ataactccgg

121 gaaaccgggg ctaataccgg atggttgttt gaaccgcatg gttcaaacat aaaaggtggc

181 ttcggctacc acttacagat ggacccgcgg cgcatwrgct tagttcggtt gacgktaays

241 gctccascaa kgcaacgatg sktagccgac ctgagasggt gatcgsmcac actgggactg

301 agacacgrcc cagactccta csggagscag cagtagggaa tcttccgcaa tggacgaaag

361 tctgacggag caacgccgcg tgagtgatga aggttttcgg atcgtaaagc tctgttgtta

421 gggaagaaca agtaccgttc gaatagggcg gtaccttgac ggtacctaac cagaaagcca

481 cggctaacta cgtgccagca gccgcggtaa tacgtaggtg gcaagcgttg tccgggaatt

541 attgggcgta aagggctcgc aggcggtttc ttaagtctga tgtgaaagcc cccggctcaa

601 ccggggaggg tcattggaaa ctggggaact tgagtgcaga agaggagagt ggaattccac

661 gtgtagcggt gaaatgcgta gagatgtgga ggaacaccag tggcgaaggc gactctctgg

721 tctgtaactg acgctgagga gcgaaagcgt ggggagcgaa caggattaga taccctggta

781 gtccacgccg taaacgatga gtgctaagtg ttagggggtt tccgcccctt agtgctgcag

841 ctaacgcatt aagcactccg cctggggagt acggtcgcaa gactgaaact caaaggaatt

901 gacgggggcc cgcacaagcg gtggagcatg tggtttaatt cgaagcaacg cgaagaacct

961 taccaggtct tgacatcctc tgacaatcct agagatagga cgtccccttt cgggggcaga

1021 gtgacaggtg gtgcatgggt tgtcgtcagc tcgtgtcgtg agatgttggg ttaagtcccg

1081 caacgagcgc aacccttgat cttagttgcc agcattcagt tgggcactct aaggtgactg

1141 ccggtgacaa accggargaa ggtggggatg acgtcaaatc atcatgcscc ttatgacctg

1201 rgctacacac gtgctacaat ggacagaaca aagggcagcg aamccgcsmg gttaagccaa

1261 tcccacaaat ctgttctcag ttcggatcgc agtctgcaac tcgactgcgt gaagctggaa

1321 tcgctagtaa tcgcggatca gcatgccgcg gtgaatacgt tcccgggcct tgtacacacc

1381 gcccgtcaca ccacgagagt ttgtaacacc cgaagtcggt gaggtaacct tttaggagcc

1441 agccgccgaa gtgacagggg

//

LOCUS PQ425640 1449 bp DNA linear BCT 04-OCT-2024

DEFINITION Bacillus subtilis strain F37 16S ribosomal RNA gene, partial sequence.

ACCESSION PQ425640

VERSION PQ425640

KEYWORDS .

SOURCE Bacillus subtilis

ORGANISM Bacillus subtilis

Bacteria; Bacillota; Bacilli; Bacillales; Bacillaceae; Bacillus.

REFERENCE 1 (bases 1 to 1449)

AUTHORS Huang,R.

TITLE Direct Submission

JOURNAL Submitted (04-OCT-2024) College of Animal Science and Technology,

Yunnan Agricultural University, Fengyuan Road No. 452, Kunming,

Yunnan 650201, China

COMMENT ##Assembly-Data-START##

Sequencing Technology :: Sanger dideoxy sequencing

##Assembly-Data-END##

FEATURES Location/Qualifiers

source 1..1449

/organism="Bacillus subtilis"

/mol_type="genomic DNA"

/strain="F37"

/db_xref="taxon:1423"

/geo_loc_name="China: Yunnan, Tengchong County"

/collection_date="May-2023"

rRNA <1..>1449

/product="16S ribosomal RNA"

ORIGIN

1 gggcggcgtg ctatacatgc agtcgagcgg acagatggga gcttgctccc tgatgttagc

61 ggcggacggg tgagtaacac gtgggtaacc tgcctgtaag actgggataa ctccgggaaa

121 ccggggctaa taccggatgg ttgtttgaac cgcatggttc aaacataaaa ggtggcttcg

181 gctaccactt acagatggac ccgcggcgca ttagctagtt ggtgaggtaa cggctcacca

241 aggcaacgat gcgtagccga cctgagaggg tgatcggcca cactgggact gagacacggc

301 ccagactcct acgggaggca gcagtaggga atcttccgca atggacgaaa gtctgacgga

361 gcaacgccgc gtgagtgatg aaggttttcg gatcgtaaag ctctgttgtt agggaagaac

421 aagtaccgtt cgaatagggc ggtaccttga cggtacctaa ccagaaagcc acggctaact

481 acgtgccagc agccgcggta atacgtaggt ggcaagcgtt gtccggaatt attgggcgta

541 aagggctcgc aggcggtttc ttaagtctga tgtgaaagcc cccggctcaa ccggggaggg

601 tcattggaaa ctggggaact tgagtgcaga agaggagagt ggaattccac gtgtagcggt

661 gaaatgcgta gagatgtgga ggaacaccag tggcgaaggc gactctctgg tctgtaactg

721 acgctgagga gcgaaagcgt ggggagcgaa caggattaga taccctggta gtccacgccg

781 taaacgatga gtgctaagtg ttagggggtt tccgcccctt agtgctgcag ctaacgcatt

841 aagcactccg cctggggagt acggtcgcaa gactgaaact caaaggaatt gacgggggcc

901 cgcacaagcg gtggagcatg tggtttaatt cgaagcaacg cgaagaacct taccaggtct

961 tgacatcctc tgacaatcct agagatagga cgtccccttc gggggcagag tgacaggtgg

1021 tgcatggttg tcgtcagctc gtgtcgtgag atgttgggtt aagtcccgca acgagcgcaa

1081 cccttgatct tagttgccag cattcagttg ggcactctaa ggtgactgcc ggtgacaaac

1141 cggaggaagg tggggatgac gtcaaatcat catgcccctt atgacctggg ctacacacgt

1201 gctacaatgg acagaacaaa gggcagcgaa accgcgaggt taagccaatc ccacaaatct

1261 gttctcagtt cggatcgcag tctgcaactc gactgcgtga agctggaatc gctagtaatc

1321 gcggatcagc atgccgcggt gaatacgttc ccgggccttg tacacaccgc ccgtcacacc

1381 acgagagttt gtaacacccg aagtcggtga ggtaaccttt taggagccag ccgccgaagt

1441 gaaccaagg

//

LOCUS PQ425641 1450 bp DNA linear BCT 04-OCT-2024

DEFINITION Bacillus subtilis strain F40 16S ribosomal RNA gene, partial sequence.

ACCESSION PQ425641

VERSION PQ425641

KEYWORDS .

SOURCE Bacillus subtilis

ORGANISM Bacillus subtilis

Bacteria; Bacillota; Bacilli; Bacillales; Bacillaceae; Bacillus.

REFERENCE 1 (bases 1 to 1450)

AUTHORS Huang,R.

TITLE Direct Submission

JOURNAL Submitted (04-OCT-2024) College of Animal Science and Technology,

Yunnan Agricultural University, Fengyuan Road No. 452, Kunming,

Yunnan 650201, China

COMMENT ##Assembly-Data-START##

Sequencing Technology :: Sanger dideoxy sequencing

##Assembly-Data-END##

FEATURES Location/Qualifiers

source 1..1450

/organism="Bacillus subtilis"

/mol_type="genomic DNA"

/strain="F40"

/db_xref="taxon:1423"

/geo_loc_name="China: Yunnan, Tengchong County"

/collection_date="May-2023"

rRNA <1..>1450

/product="16S ribosomal RNA"

ORIGIN

1 gggggggggt gctatacatg cagtcgagcg gacagatggg agcttgctcc ctgatgttag

61 cggcggacgg gtgagtaaca cgtgggtaac ctgcctgtaa gactgggata actccgggaa

121 accggggcta ataccggatg gttgtttgaa ccgcatggtt caaacataaa aggtggcttc

181 ggctaccact tacagatgga cccgcggcgc attagctagt tggtgaggta acggctcacc

241 aaggcaacga tgcgtagccg acctgagagg gtgatcggcc acactgggac tgagacacgg

301 cccagactcc tacgggaggc agcagtaggg aatcttccgc aatggacgaa agtctgacgg

361 agcaacgccg cgtgagtgat gaaggttttc ggatcgtaaa gctctgttgt tagggaagaa

421 caagtaccgt tcgaataggg cggtaccttg acggtaccta accagaaagc cacggctaac

481 tacgtgccag cagccgcggt aatacgtagg tggcaagcgt tgtccggaat tattgggcgt

541 aaagggctcg caggcggttt cttaagtctg atgtgaaagc ccccggctca accggggagg

601 gtcattggaa actggggaac ttgagtgcag aagaggagag tggaattcca cgtgtagcgg

661 tgaaatgcgt agagatgtgg aggaacacca gtggcgaagg cgactctctg gtctgtaact

721 gacgctgagg agcgaaagcg tggggagcga acaggattag ataccctggt agtccacgcc

781 gtaaacgatg agtgctaagt gttagggggt ttccgcccct tagtgctgca gctaacgcat

841 taagcactcc gcctggggag tacggtcgca agactgaaac tcaaaggaat tgacgggggc

901 ccgcacaagc ggtggagcat gtggtttaat tcgaagcaac gcgaagaacc ttaccaggtc

961 ttgacatcct ctgacaatcc tagagatagg acgtcccctt cgggggcaga gtgacaggtg

1021 gtgcatggtt gtcgtcagct cgtgtcgtga gatgttgggt taagtcccgc aacgagcgca

1081 acccttgatc ttagttgcca gcattcagtt gggcactcta aggtgactgc cggtgacaaa

1141 ccggaggaag gtggggatga cgtcaaatca tcatgcccct tatgacctgg gctacacacg

1201 tgctacaatg gacagaacaa agggcagcga aaccgcgagg ttaagccaat cccacaaatc

1261 tgttctcagt tcggatcgca gtctgcaact cgactgcgtg aagctggaat cgctagtaat

1321 cgcggatcag catgccgcgg tgaatacgtt cccgggcctt gtacacaccg cccgtcacac

1381 cacgagagtt tgtaacaccc gaagtcggtg aggtaacctt ttaggagcca gccgccgaag

1441 tgatcagagg

//

LOCUS PQ425642 1448 bp DNA linear BCT 04-OCT-2024

DEFINITION Bacillus subtilis strain F44 16S ribosomal RNA gene, partial sequence.

ACCESSION PQ425642

VERSION PQ425642

KEYWORDS .

SOURCE Bacillus subtilis

ORGANISM Bacillus subtilis

Bacteria; Bacillota; Bacilli; Bacillales; Bacillaceae; Bacillus.

REFERENCE 1 (bases 1 to 1448)

AUTHORS Huang,R.

TITLE Direct Submission

JOURNAL Submitted (04-OCT-2024) College of Animal Science and Technology,

Yunnan Agricultural University, Fengyuan Road No. 452, Kunming,

Yunnan 650201, China

COMMENT ##Assembly-Data-START##

Sequencing Technology :: Sanger dideoxy sequencing

##Assembly-Data-END##

FEATURES Location/Qualifiers

source 1..1448

/organism="Bacillus subtilis"

/mol_type="genomic DNA"

/strain="F44"

/db_xref="taxon:1423"

/geo_loc_name="China: Yunnan, Tengchong County"

/collection_date="May-2023"

rRNA <1..>1448

/product="16S ribosomal RNA"

ORIGIN

1 agggcggcgg ctatacatgc agtcgagcgg acagatggga gcttgctccc tgatgttagc

61 ggcggacggg tgagtaacac gtgggtaacc tgcctgtaag actgggataa ctccgggaaa

121 ccggggctaa taccggatgg ttgtttgaac cgcatggttc aaacataaaa ggtggcttcg

181 gctaccactt acagatggac ccgcggcgca ttagctagtt ggtgaggtaa cggctcacca

241 aggcaacgat gcgtagccga cctgagaggg tgatcggcca cactgggact gagacacggc

301 ccagactcct acgggaggca gcagtaggga atcttccgca atggacgaaa gtctgacgga

361 gcaacgccgc gtgagtgatg aaggttttcg gatcgtaaag ctctgttgtt agggaagaac

421 aagtaccgtt cgaatagggc ggtaccttga cggtacctaa ccagaaagcc acggctaact

481 acgtgccagc agccgcggta atacgtaggt ggcaagcgtt gtccggaatt attgggcgta

541 aagggctcgc aggcggtttc ttaagtctga tgtgaaagcc cccggctcaa ccggggaggg

601 tcattggaaa ctggggaact tgagtgcaga agaggagagt ggaattccac gtgtagcggt

661 gaaatgcgta gagatgtgga ggaacaccag tggcgaaggc gactctctgg tctgtaactg

721 acgctgagga gcgaaagcgt ggggagcgaa caggattaga taccctggta gtccacgccg

781 taaacgatga gtgctaagtg ttagggggtt tccgcccctt agtgctgcag ctaacgcatt

841 aagcactccg cctggggagt acggtcgcaa gactgaaact caaaggaatt gacgggggcc

901 cgcacaagcg gtggagcatg tggtttaatt cgaagcaacg cgaagaacct taccaggtct

961 tgacatcctc tgacaatcct agagatagga cgtccccttc gggggcagag tgacaggtgg

1021 tgcatggttg tcgtcagctc gtgtcgtgag atgttgggtt aagtcccgca acgagcgcaa

1081 cccttgatct tagttgccag cattcagttg ggcactctaa ggtgactgcc ggtgacaaac

1141 cggaggaagg tggggatgac gtcaaatcat catgcccctt atgacctggg ctacacacgt

1201 gctacaatgg acagaacaaa gggcagcgaa accgcgaggt taagccaatc ccacaaatct

1261 gttctcagtt cggatcgcag tctgcaactc gactgcgtga agctggaatc gctagtaatc

1321 gcggatcagc atgccgcggt gaatacgttc ccgggccttg tacacaccgc ccgtcacacc

1381 acgagagttt gtaacacccg aagtcggtga ggtaaccttt taggagccag ccgccgaagt

1441 gacagatg

//

LOCUS PQ425643 1448 bp DNA linear BCT 04-OCT-2024

DEFINITION Bacillus subtilis strain F45 16S ribosomal RNA gene, partial sequence.

ACCESSION PQ425643

VERSION PQ425643

KEYWORDS .

SOURCE Bacillus subtilis

ORGANISM Bacillus subtilis

Bacteria; Bacillota; Bacilli; Bacillales; Bacillaceae; Bacillus.

REFERENCE 1 (bases 1 to 1448)

AUTHORS Huang,R.

TITLE Direct Submission

JOURNAL Submitted (04-OCT-2024) College of Animal Science and Technology,

Yunnan Agricultural University, Fengyuan Road No. 452, Kunming,

Yunnan 650201, China

COMMENT ##Assembly-Data-START##

Sequencing Technology :: Sanger dideoxy sequencing

##Assembly-Data-END##

FEATURES Location/Qualifiers

source 1..1448

/organism="Bacillus subtilis"

/mol_type="genomic DNA"

/strain="F45"

/db_xref="taxon:1423"

/geo_loc_name="China: Yunnan, Tengchong County"

/collection_date="May-2023"

rRNA <1..>1448

/product="16S ribosomal RNA"

ORIGIN

1 agggcgggtg ctatacatgc aagtcgagcg gacagatggg agcttgctcc ctgatgttag

61 cggcggacgg gtgagtaaca cgtgggtaac ctgcctgtaa gactgggata actccgggaa

121 accggggcta ataccggatg gttgtttgaa ccgcatggtt caaacataaa aggtggcttc

181 ggctaccact tacagatgga cccgcggcgc attagctagt tggtgaggta acggctcacc

241 aaggcaacga tgcgtagccg acctgagagg gtgatcggcc acactgggac tgagacacgg

301 cccagactcc tacgggaggc agcagtaggg aatcttccgc aatggacgaa agtctgacgg

361 agcaacgccg cgtgagtgat gaaggttttc ggatcgtaaa gctctgttgt tagggaagaa

421 caagtaccgt tcgaataggg cggtaccttg acggtaccta accagaaagc cacggctaac

481 tacgtgccag cagccgcggt aatacgtagg tggcaagcgt tgtccggaat tattgggcgt

541 aaagggctcg caggcggttt cttaagtctg atgtgaaagc ccccggctca accggggagg

601 gtcattggaa actggggaac ttgagtgcag aagaggagag tggaattcca cgtgtagcgg

661 tgaaatgcgt agagatgtgg aggaacacca gtggcgaagg cgactctctg gtctgtaact

721 gacgctgagg agcgaaagcg tggggagcga acaggattag ataccctggt agtccacgcc

781 gtaaacgatg agtgctaagt gttagggggt ttccgcccct tagtgctgca gctaacgcat

841 taagcactcc gcctggggag tacggtcgca agactgaaac tcaaaggaat tgacgggggc

901 ccgcacaagc ggtggagcat gtggtttaat tcgaagcaac gcgaagaacc ttaccaggtc

961 ttgacatcct ctgacaatcc tagagatagg acgtcccctt cgggggcaga gtgacaggtg

1021 gtgcatggtt gtcgtcagct cgtgtcgtga gatgttgggt taagtcccgc aacgagcgca

1081 acccttgatc ttagttgcca gcattcagtt gggcactcta aggtgactgc cggtgacaaa

1141 ccggaggaag gtggggatga cgtcaaatca tcatgcccct tatgacctgg gctacacacg

1201 tgctacaatg gacagaacaa agggcagcga aaccgcgagg ttaagccaat cccacaaatc

1261 tgttctcagt tcggatcgca gtctgcaact cgactgcgtg aagctggaat cgctagtaat

1321 cgcggatcag catgccgcgg tgaatacgtt cccgggcctt gtacacaccg cccgtcacac

1381 cacgagagtt tgtaacaccc gaagtcggtg aggtaacctt taggagccag ccgccgaagt

1441 gacaaaga

//

LOCUS PQ425644 1448 bp DNA linear BCT 04-OCT-2024

DEFINITION Bacillus subtilis strain F46 16S ribosomal RNA gene, partial sequence.

ACCESSION PQ425644

VERSION PQ425644

KEYWORDS .

SOURCE Bacillus subtilis

ORGANISM Bacillus subtilis

Bacteria; Bacillota; Bacilli; Bacillales; Bacillaceae; Bacillus.

REFERENCE 1 (bases 1 to 1448)

AUTHORS Huang,R.

TITLE Direct Submission

JOURNAL Submitted (04-OCT-2024) College of Animal Science and Technology,

Yunnan Agricultural University, Fengyuan Road No. 452, Kunming,

Yunnan 650201, China

COMMENT ##Assembly-Data-START##

Sequencing Technology :: Sanger dideoxy sequencing

##Assembly-Data-END##

FEATURES Location/Qualifiers

source 1..1448

/organism="Bacillus subtilis"

/mol_type="genomic DNA"

/strain="F46"

/db_xref="taxon:1423"

/geo_loc_name="China: Yunnan, Tengchong County"

/collection_date="May-2023"

rRNA <1..>1448

/product="16S ribosomal RNA"

ORIGIN

1 ggggggggtg ctatacatgc agtcgagcgg acagatggga gcttgctccc tgatgttagc

61 ggcggacggg tgagtaacac gtgggtaacc tgcctgtaag actgggataa ctccgggaaa

121 ccggggctaa taccggatgg ttgtttgaac cgcatggttc aaacataaaa ggtggcttcg

181 gctaccactt acagatggac ccgcggcgca ttagctagtt ggtgaggtaa cggctcacca

241 aggcaacgat gcgtagccga cctgagaggg tgatcggcca cactgggact gagacacggc

301 ccagactcct acgggaggca gcagtaggga atcttccgca atggacgaaa gtctgacgga

361 gcaacgccgc gtgagtgatg aaggttttcg gatcgtaaag ctctgttgtt agggaagaac

421 aagtaccgtt cgaatagggc ggtaccttga cggtacctaa ccagaaagcc acggctaact

481 acgtgccagc agccgcggta atacgtaggt ggcaagcgtt gtccggaatt attgggcgta

541 aagggctcgc aggcggtttc ttaagtctga tgtgaaagcc cccggctcaa ccggggaggg

601 tcattggaaa ctggggaact tgagtgcaga agaggagagt ggaattccac gtgtagcggt

661 gaaatgcgta gagatgtgga ggaacaccag tggcgaaggc gactctctgg tctgtaactg

721 acgctgagga gcgaaagcgt ggggagcgaa caggattaga taccctggta gtccacgccg

781 taaacgatga gtgctaagtg ttagggggtt tccgcccctt agtgctgcag ctaacgcatt

841 aagcactccg cctggggagt acggtcgcaa gactgaaact caaaggaatt gacgggggcc

901 cgcacaagcg gtggagcatg tggtttaatt cgaagcaacg cgaagaacct taccaggtct

961 tgacatcctc tgacaatcct agagatagga cgtccccttc gggggcagag tgacaggtgg

1021 tgcatggttg tcgtcagctc gtgtcgtgag atgttgggtt aagtcccgca acgagcgcaa

1081 cccttgatct tagttgccag cattcagttg ggcactctaa ggtgactgcc ggtgacaaac

1141 cggaggaagg tggggatgac gtcaaatcat catgcccctt atgacctggg ctacacacgt

1201 gctacaatgg acagaacaaa gggcagcgaa accgcgaggt taagccaatc ccacaaatct

1261 gttctcagtt cggatcgcag tctgcaactc gactgcgtga agctggaatc gctagtaatc

1321 gcggatcagc atgccgcggt gaatacgttc ccgggccttg tacacaccgc ccgtcacacc

1381 acgagagttt gtaacacccg aagtcggtga ggtaaccttt taggagccag ccgccgaagt

1441 gacagagt

//

LOCUS PQ425645 1468 bp DNA linear BCT 04-OCT-2024

DEFINITION Bacillus subtilis strain F50 16S ribosomal RNA gene, partial sequence.

ACCESSION PQ425645

VERSION PQ425645

KEYWORDS .

SOURCE Bacillus subtilis

ORGANISM Bacillus subtilis

Bacteria; Bacillota; Bacilli; Bacillales; Bacillaceae; Bacillus.

REFERENCE 1 (bases 1 to 1468)

AUTHORS Huang,R.

TITLE Direct Submission

JOURNAL Submitted (04-OCT-2024) College of Animal Science and Technology,

Yunnan Agricultural University, Fengyuan Road No. 452, Kunming,

Yunnan 650201, China

COMMENT ##Assembly-Data-START##

Sequencing Technology :: Sanger dideoxy sequencing

##Assembly-Data-END##

FEATURES Location/Qualifiers

source 1..1468

/organism="Bacillus subtilis"

/mol_type="genomic DNA"

/strain="F50"

/db_xref="taxon:1423"

/geo_loc_name="China: Yunnan, Tengchong County"

/collection_date="May-2023"

rRNA <1..>1468

/product="16S ribosomal RNA"

ORIGIN

1 gcgtgcgggt gctatacatg cagtcgagcg gacagatggg agcttgctcc ctgatgttag

61 cggcggacgg gtgagtaaca cgtgggtaac ctgcctgtaa gactgggata actccgggaa

121 accggggcta ataccggatg gttgtttgaa ccgcatggtt caaacataaa aggtggcttc

181 ggctaccact tacagatgga cccgcggcgc attagctagt tgrtgagkta atcggctcca

241 gaccaaggcc amcgaattgc gtagccggac cttgagaggg tgatcggstc acactggrac

301 tgagaacacg kcccagactc ctacsggagg cagcagtaag ggaatcttcc gcaatggacg

361 aaagtctgac ggagcaacgc cgcgtgagtg atgaaggttt tcggatcgta aagctctgtt

421 gttagggaag aacaagtacc gttcgaatag ggcggtacct tgacggtacc taaccagaaa

481 gccacggcta actacgtgcc agcagccgcg gtaatacgta ggtggcaagc gttgtccgga

541 attattgggc gtaaagggct cgcaggcggt ttcttaagtc tgatgtgaaa gcccccggct

601 caaccgggga gggtcattgg aaactgggga acttgagtgc agaagaggag agtggaattc

661 cacgtgtagc ggtgaaatgc gtagagatgt ggaggaacac cagtggcgaa ggcgactctc

721 tggtctgtaa ctgacgctga ggagcgaaag cgtggggagc gaacaggatt agataccctg

781 gtagtccacg ccgtaaacga tgagtgctaa gtgttagggg gtttccgccc cttagtgctg

841 cagctaacgc attaagcact ccgcctgggg agtacggtcg caagactgaa actcaaagga

901 attgacgggg gcccgcacaa gcggtggagc atgtggttta attcgaagca acgcgaagaa

961 ccttaccagg tcttgacatc ctctgacaat cctagagata ggacgtcccc ttcgggggca

1021 gagtgacagg tggtgcatgg gttgtcgtca gctcgtgtcg tgagatgttg ggttaagtcc

1081 cgcaacgagc gcamccyttg atcttagttg ccagcattca gttgggcact ctaaggtgac

1141 tgccggtgac aaaccggagg aaggtgggga atgacgtcaa atcatcatgc cccttatgac

1201 ctkggcttac acacgtgctt acawtgggam mgaacmaakg gcagcgaaac cgcgcaggtt

1261 aagccaatcc cacaaatctg ttctcagttc ggatcgcagt ctgcaactcg actgcgtgaa

1321 gctggaatcg ctagtaatcg cggatcagca tgccgcggtg aatacgttcc cgggccttgt

1381 acacaccgcc cgtcacacca cgagagtttg taacacccga agtcggtgag gtaacctttt

1441 aggagccagc cgccgaagtg acagatga

//

LOCUS PQ425646 1437 bp DNA linear BCT 04-OCT-2024

DEFINITION Pantoea agglomerans strain F53 16S ribosomal RNA gene, partial sequence.

ACCESSION PQ425646

VERSION PQ425646

KEYWORDS .

SOURCE Pantoea agglomerans

ORGANISM Pantoea agglomerans

Bacteria; Pseudomonadota; Gammaproteobacteria; Enterobacterales;

Erwiniaceae; Pantoea; Pantoea agglomerans group.

REFERENCE 1 (bases 1 to 1437)

AUTHORS Huang,R.

TITLE Direct Submission

JOURNAL Submitted (04-OCT-2024) College of Animal Science and Technology,

Yunnan Agricultural University, Fengyuan Road No. 452, Kunming,

Yunnan 650201, China

COMMENT ##Assembly-Data-START##

Sequencing Technology :: Sanger dideoxy sequencing

##Assembly-Data-END##

FEATURES Location/Qualifiers

source 1..1437

/organism="Pantoea agglomerans"

/mol_type="genomic DNA"

/strain="F53"

/db_xref="taxon:549"

/geo_loc_name="China: Yunnan, Tengchong County"

/collection_date="May-2023"

rRNA <1..>1437

/product="16S ribosomal RNA"

ORIGIN

1 gcggtgcgca gctaccatgc agtcgaacgg tagcacagag agcttgctct tgggtgacga

61 gtggcggacg ggtgagtaat gtctggggat ctgcctgaca gagggggata actactggaa

121 acggtagcta ataccgcata acctcgcaag agcaaagagg gggaccttcg ggcctctcgc

181 tgtcagatga acccagatgg gattagctag taggtggggt aatggctcac ctaggcgacg

241 atccctagct ggtctgagag gatgaccagc cacactggaa ctgagacacg gtccagactc

301 ctacgggagg cagcagtggg gaatattgca caatgggcgc aagcctgatg cagccatgcc

361 gcgtgtatga agaaggcctt cgggttgtaa agtactttca gcggggagga aggtgttgag

421 gttaataacc tcagcaattg acgttacccg cagaagaagc accggctaac tccgtgccag

481 cagccgcggt aatacggagg gtgcaagcgt taatcggaat tactgggcgt aaagcgcacg

541 caggcggtct gtcaagtcag atgtgaaatc cccgggctta acctgggaac tgcatttgaa

601 actggcaggc tagagtcttg tagagggggg tagaattcca ggtgtagcgg tgaaatgcgt

661 agagatctgg aggaataccg gtggcgaagg cggccccctg gacaaagact gacgctcagg

721 tgcgaaagcg tggggagcaa acaggattag ataccctggt agtccacgcc gtaaacgatg

781 tcgacttgga ggttgttccc ttgaggagtg gcttccggag ctaacgcgtt aagtcgaccg

841 cctggggagt acggccgcaa ggttaaaact caaatgaatt gacgggggcc cgcacaagcg

901 gtggagcatg tggtttaatt cgatgcaacg cgaagaacct tacctactct tgacatccag

961 rgaatttggc agagatgcct tggtgccttc gggaaccgtg agacaggtgc tgcatggctg

1021 tcgtcagctc gtgttgtgaa atgttgggtt aagtcccgca acgagcgcaa cccttatcct

1081 ttgttgccag cacgtaatgg tgggaactca aaggagactg ccggtgataa accggaggaa

1141 ggtggggatg acgtcaagtc atcatggccc ttacgagtag ggctacacac gtgctacaat

1201 ggcgcataca aagagaagcg acctcgcgag agcaagcgga cctcataaag tgcgtcgtag

1261 tccggatcgg agtctgcaac tcgactccgt gaagtcggaa tcgctagtaa tcgtggatca

1321 gaatgccacg gtgaatacgt tcccgggcct tgtacacacc gcccgtcaca ccatgggagt

1381 gggttgcaaa agaagtaggt agcttaacct tcgggagggc gctaccactt tgataag

//

LOCUS PQ425647 1470 bp DNA linear BCT 04-OCT-2024

DEFINITION Bacillus sp. (in: firmicutes) strain F56 16S ribosomal RNA gene, partial sequence.

ACCESSION PQ425647

VERSION PQ425647

KEYWORDS .

SOURCE Bacillus sp. (in: firmicutes)

ORGANISM Bacillus sp. (in: firmicutes)

Bacteria; Bacillota; Bacilli; Bacillales; Bacillaceae; Bacillus.

REFERENCE 1 (bases 1 to 1470)

AUTHORS Huang,R.

TITLE Direct Submission

JOURNAL Submitted (04-OCT-2024) College of Animal Science and Technology,

Yunnan Agricultural University, Fengyuan Road No. 452, Kunming,

Yunnan 650201, China

COMMENT ##Assembly-Data-START##

Sequencing Technology :: Sanger dideoxy sequencing

##Assembly-Data-END##

FEATURES Location/Qualifiers

source 1..1470

/organism="Bacillus sp. (in: firmicutes)"

/mol_type="genomic DNA"

/strain="F56"

/db_xref="taxon:1409"

/geo_loc_name="China: Yunnan, Tengchong County"

/collection_date="May-2023"

rRNA <1..>1470

/product="16S ribosomal RNA"

ORIGIN

1 ccgaatggcg gggtgctata catgcaagtc gagcggacag atgggagctt gctccctgat

61 gttagcggcg gacgggtgag taacacgtgg gtaacctgcc tgtaagactg ggataactcc

121 gggaaaccgg ggctaatacc ggatggttgt ttgaaccgca tggttcaaac ataaaaggtg

181 gcttcggcta ccacttacag atggacccgc ggcgcattag ctaggttcgg tgaagstaac

241 cggstcaccc awgscaatcg atgcgtagcc gacctgagak gstgatcgkc cacactggga

301 ctgagacacg kcccagactc ctacgggagg cagcagtagg gaatcttccg caatggacga

361 aagtctgacs gagcaacgcc gcgtgagtga tgaaggtttt cggatcgtaa agctctgttg

421 ttagggaaga acaagtaccg ttcgaatagg gcggtacctt gacggtacct aacccagaaa

481 gccacggcta actacgtgcc agcagccgcg gtaatacgta ggtggcaagc gttgtccgga

541 attattgggc gtaaagggct cgcaggcggt ttcttaagtc tgatgtgaaa gcccccggct

601 caaccgggga gggtcattgg aaactgggga acttgagtgc agaagaggag agtggaattc

661 cacgtgtagc ggtgaaatgc gtagagatgt ggaggaacac cagtggcgaa ggcgactctc

721 tggtctgtaa ctgacgctga ggagcgaaag cgtggggagc gaacaggatt agataccctg

781 gtagtccacg ccgtaaacga tgagtgctaa gtgttagggg gtttccgccc cttagtgctg

841 cagctaacgc attaagcact ccgcctgggg agtacggtcg caagactgaa actcaaagga

901 attgacgggg gcccgcacaa gcggtggagc atgtggttta attcgaagca acgcgaagaa

961 ccttaccagg tcttgacatc ctctgacaat cctagagata ggacgtcccc ttcgggggca

1021 gagtgacagg tggtgcatgg gttgtcgtca gctcgtgtcg tgagatgttg ggttaagtcc

1081 cgcaacgagc gcaacccttg atcttagttg ccagcattca gttgggcacc tctaaggtga

1141 ctgccggtga caaaccggag gaaggtgggg gatgacgtcm aatcatcatg ccccttatgr

1201 mctgggctac acacgtgcta caattgraca gaacaamggg cagcgaamcs gcgaagggtt

1261 aagscaatcc cacaaatctg ttctcagttc ggatcgcagt ctgcaactcg actgcgtgaa

1321 gctggaatcg ctagtaatcg cggatcagca tgccgcggtg aatacgttcc cgggccttgt

1381 acacaccgcc cgtcacacca cgagagtttg taacacccga agtcggtgag gtaacctttt

1441 aggagccagc cgccgaaggt gacagaatgt

//

LOCUS PQ425648 1459 bp DNA linear BCT 04-OCT-2024

DEFINITION Bacillus subtilis strain F60 16S ribosomal RNA gene, partial sequence.

ACCESSION PQ425648

VERSION PQ425648

KEYWORDS .

SOURCE Bacillus subtilis

ORGANISM Bacillus subtilis

Bacteria; Bacillota; Bacilli; Bacillales; Bacillaceae; Bacillus.

REFERENCE 1 (bases 1 to 1459)

AUTHORS Huang,R.

TITLE Direct Submission

JOURNAL Submitted (04-OCT-2024) College of Animal Science and Technology,

Yunnan Agricultural University, Fengyuan Road No. 452, Kunming,

Yunnan 650201, China

COMMENT ##Assembly-Data-START##

Sequencing Technology :: Sanger dideoxy sequencing

##Assembly-Data-END##

FEATURES Location/Qualifiers

source 1..1459

/organism="Bacillus subtilis"

/mol_type="genomic DNA"

/strain="F60"

/db_xref="taxon:1423"

/geo_loc_name="China: Yunnan, Tengchong County"

/collection_date="May-2023"

rRNA <1..>1459

/product="16S ribosomal RNA"

ORIGIN

1 aggcagtgcg gcgtgctata catgcagtcg agcggacaga tgggagcttg ctccctgatg

61 ttagcggcgg acgggtgagt aacacgtggg taacctgcct gtaagactgg gataactccg

121 ggaaaccggg gctaataccg gatggttgtt tgaaccgcat ggttcaaaca taaaaggtgg

181 cttcggctac cacttacaga tggacccgcg gmgcmttagc tagttggtga ggtawcggct

241 cacccaaggc aacgatgcgt agccgacctg rragggtgat csrccacact gggactgaga

301 cacgkcccag actcctacgg gaggccagca gtagggaatc ttccgcaatg gacgaaagtc

361 tgacggagca acgccgcgtg agtgatgaag gttttcggat cgtaaagctc tgttgttagg

421 gaagaacaag taccgttcga atagggcggt accttgacgg tacctaacca gaaagccacg

481 gctaactacg tgccagcagc cgcggtaata cgtaggtggc aagcgttgtc cggaattatt

541 gggcgtaaag ggctcgcagg cggtttctta agtctgatgt gaaagccccc ggctcaaccg

601 gggagggtca ttggaaactg gggaacttga gtgcagaaga ggagagtgga attccacgtg

661 tagcggtgaa atgcgtagag atgtggagga acaccagtgg cgaaggcgac tctctggtct

721 gtaactgacg ctgaggagcg aaagcgtggg gagcgaacag gattagatac cctggtagtc

781 cacgccgtaa acgatgagtg ctaagtgtta gggggtttcc gccccttagt gctgcagcta

841 acgcattaag cactccgcct ggggagtacg gtcgcaagac tgaaactcaa aggaattgac

901 gggggcccgc acaagcggtg gagcatgtgg tttaattcga agcaacgcga agaaccttac

961 caggtcttga catcctctga caatcctaga gataggacgt cctccttcgg gggcagagtg

1021 acaggtggtg catgggttgt cgtcagctcg tgtcgtgaga tgttgggkta agtcccgcaa

1081 cgagcgcaac ccttgatctt agttgccagc attcagttgg gcacctctaa ggtgactgsc

1141 ggtgacaaac cggaggaagg tkgggatgac gtcaaatcat catgcccstt atgacctggg

1201 ctacacacgt gcttacaatk gacrgamcaa agggcagcsw aamckckagg ttaagccaat

1261 cccacaaatc tgttctcagt tcggatcgca gtctgcaact cgactgcgtg aagctggaat

1321 cgctagtaat cgcggatcag catgccgcgg tgaatacgtt cccgggcctt gtacacaccg

1381 cccgtcacac cacgagagtt tgtaacaccc gaagtcggtg aggtaacctt ttaggagcca

1441 gccgccgaag tgacagagg
